# Comparative (Meta)genomic Analysis and Ecological Profiling of Human Gut-Specific Bacteriophage φB124-14

**DOI:** 10.1371/journal.pone.0035053

**Published:** 2012-04-25

**Authors:** Lesley A. Ogilvie, Jonathan Caplin, Cinzia Dedi, David Diston, Elizabeth Cheek, Lucas Bowler, Huw Taylor, James Ebdon, Brian V. Jones

**Affiliations:** 1 Centre for Biomedical and Health Science Research, School of Pharmacy and Biomolecular Sciences, University of Brighton, Brighton, United Kingdom; 2 School of Environment and Technology, University of Brighton, Brighton, United Kingdom; 3 School of Computing, Engineering and Mathematics, University of Brighton, Brighton, United Kingdom; 4 Sussex Proteomics Centre, University of Sussex, Brighton, United Kingdom; J. Craig Venter Institute, United States of America

## Abstract

Bacteriophage associated with the human gut microbiome are likely to have an important impact on community structure and function, and provide a wealth of biotechnological opportunities. Despite this, knowledge of the ecology and composition of bacteriophage in the gut bacterial community remains poor, with few well characterized gut-associated phage genomes currently available. Here we describe the identification and in-depth (meta)genomic, proteomic, and ecological analysis of a human gut-specific bacteriophage (designated φB124-14). In doing so we illuminate a fraction of the biological dark matter extant in this ecosystem and its surrounding eco-genomic landscape, identifying a novel and uncharted bacteriophage gene-space in this community. φB124-14 infects only a subset of closely related gut-associated *Bacteroides fragilis* strains, and the circular genome encodes functions previously found to be rare in viral genomes and human gut viral metagenome sequences, including those which potentially confer advantages upon phage and/or host bacteria. Comparative genomic analyses revealed φB124-14 is most closely related to φB40-8, the only other publically available *Bacteroides sp*. phage genome, whilst comparative metagenomic analysis of both phage failed to identify any homologous sequences in 136 non-human gut metagenomic datasets searched, supporting the human gut-specific nature of this phage. Moreover, a potential geographic variation in the carriage of these and related phage was revealed by analysis of their distribution and prevalence within 151 human gut microbiomes and viromes from Europe, America and Japan. Finally, ecological profiling of φB124-14 and φB40-8, using both gene-centric alignment-driven phylogenetic analyses, as well as alignment-free gene-independent approaches was undertaken. This not only verified the human gut-specific nature of both phage, but also indicated that these phage populate a distinct and unexplored ecological landscape within the human gut microbiome.

## Introduction

The human gut harbours a diverse microbial community which in turn plays host to a variety of mobile genetic elements (MGE) and bacteriophages, forming the gut mobile metagenome [1]–[4]. The role of this flexible gene pool in the development and functioning of the gut microbial community remains largely unexplored, yet there is emerging evidence that this mobile metagenome reflects the co-evolution of host and microbe in this community, and that some MGE may be unique to or enriched within this ecosystem [1], [4]–[8].

Identification and characterization of such elements will provide much insight into fundamental aspects of development and functioning of the gut microbiota, and provide the raw material for the development of novel molecular tools. Furthermore, MGE comprising the human gut mobile metagenome are also likely to encode a range of functions of biotechnological or pharmaceutical interest [9]. Bacteriophages in particular have the potential to influence community structure and function [10]–[15], and are regarded to be of considerable biotechnological value, exemplified by the growing interest in their use as novel and highly selective therapeutic agents (for review see [16]). Initial studies of the gut virome have already provided evidence of distinct viral population dynamics and gene content in this ecosystem, with a dominance of apparently temperate phage and a relative lack of the predator-prey phage-host relationship commonly observed in other microbial communities [6].

Through selective elimination of species within the gut microbiota, phage may alter community function, metabolic output and subsequently impact on host health [17]–[19]. Furthermore, there is also scope for the direct interaction of bacteriophage with the host immune system, which may be important in the pathogenesis of some gut related disorders [17]. The observation that dense bacteriophage populations are associated with the gut mucosa, and numbers are elevated in patients with Crohn's disease, emphasizes the possible role of these bacterial viruses in community function, interaction with the host, and disease pathogenesis [19].

The characterisation of bacteriophage specific to the human gut is also of considerable interest for the development of microbial source tracking tools (MST), which permit determination of faecal source in surface and ground waters [20]–[24]. Faecal contamination of surface waters poses a major risk to public health, and bacteriophages specific to human faecal indicator bacteria (and the human gut microbiome) have already been successfully employed as water quality indicators that can specifically identify pollution originating from human sources [20]–[22], [25], [26]. Bacteriophage offer numerous advantages in these applications and are not only thought to persist longer in the environment than host bacteria but can often be found in higher numbers making them a more sensitive source tracking tool [22]. In particular, the development of rapid and sensitive culture-independent methods for detection of human faecal indicator phage, directly in environmental samples, offers significant advantages over classic culture-based approaches, and there is presently much interest in developing and implementing such strategies [27].

However, the development of culture-independent phage-based MST tools, along with our improved understanding of bacteriophage in the gut community, is hindered by the lack of well-characterized bacteriophage with defined host-ranges and available genome sequences, which infect prominent and important species of human gut bacteria. A prime example is the availability of only one complete *Bacteroides spp.* phage genome sequence in public databases (as of Oct 2011), despite the prominence and importance of this group of bacteria in the human gut microbiome [28], [29].

We have previously isolated bacteriophage infecting the human faecal indicator bacteria *Bacteroides sp*. GB-124 from municipal wastewaters, and found these to be present in human faecal samples, but absent from faecal samples derived from a wide range of domestic and wild animals, as well as from the general environment, strongly suggesting these phage are human gut-specific [20]. Because of the apparent gut-specific nature of these phage, and the growing evidence of their usefulness as MST, in-depth genomic characterization would not only begin to address the current lack of knowledge regarding gut-associated bacteriophage (and *Bacteroides* phage in particular), but would also provide the genetic information required for development of culture-independent MST.

This motivated us to undertake an in-depth characterization of one such phage designated φB124-14. This phage was selected as it not only appears to be representative of a morphologically and phenotypically homogenous group of human-specific phages, but also displayed greater environmental stability than other phage tested (particularly in terms of UV resistance), suggesting an excellent environmental “half-life” (D. Diston Jan 2010, pers. comm.). Here we have characterized the host range, complete genome sequence and proteome of φB124-14. Using comparative metagenomic analysis and genome signature-based approaches we subsequently examined its ecological profile in relation to 611 other bacteriophage genomes available on GenBank, as well as human gut-specific viral metagenomes [6].

Overall, these investigations support the human gut specific nature of φB124-14 and indicate that this phage occupies a distinct and largely unexplored ecological landscape within the human gut microbiome. We also increase the available number of well-characterized genomes of bacteriophage infecting prominent members of the human gut microbiota. This will not only enhance our fundamental understanding of this important microbial ecosystem, but will facilitate the development of sensitive and rapid culture-independent MST tools.

## Results and Discussion

### φB124-14 physical characteristics and host range

**Figure 1:**
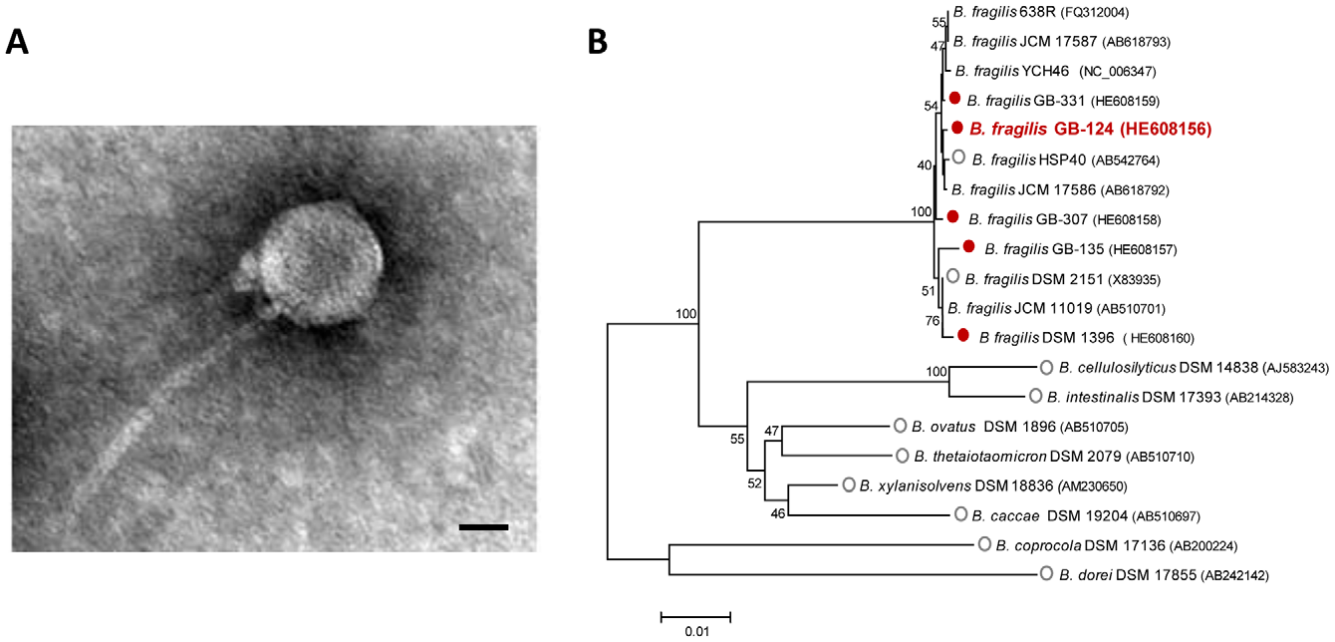
Physical structure and host range of ΦB124-14. **A.** Transmission electron micrograph of ΦB124-14 showing phage capsid composed of an icosahedral head and a non-contractile tail. Magnification 50,000×. Scale Bar, 20 nm. **B.** Phylogenetic characterisation of *B. fragilis* φB124-14 host strains. Consensus maximum likelihood trees were constructed from 16S rRNA gene sequences, with 1000 bootstrap resamplings using MEGA v5. Bootstrap values of 40 or greater are shown adjacent to respective nodes. Accession numbers for bacterial 16S sequences are given in brackets following species names on the tree. The ability of φB124-14 to replicate in a particular host species was tested in standard agar overlay assays, in which replication of φB124-14 in a particular host was indicated by production of plaques in bacterial lawns. Species tested in host range assays are denoted by open or filled circles. Filled red circles indicate strains which support φB124-14 replication, and open grey circles indicate strains in which φB124 did not replicate.

Transmission Electron Microscopy (TEM) shows φB124-14 has a binary morphology with an icosahedral head and a non-contractile tail (Figure 1A), placing it in the *Caudoviriale* order, *Siphoviridae* family [30]. The phage produces small (0.7 mm ±0.3), clear plaques on a lawn of the original host *Bacteroides sp*. GB-124. Structural dimensions are similar to the *B. fragilis* faecal pollution indicator phage B40-8 (φB40-8; also referred to as phage ATCC 51477-B1; GenBank accession no. FJ008913.1) [31], with tail length of 162 nm ±21, tail diameter of 13.6 nm ±1.6, and a slightly smaller head diameter measurement of 49.8 nm ±3.9 (versus φB40-8 measurements of 60 ± 4.0 nm). The morphology of the φB124-14 capsid is in keeping with metagenomic surveys of human gut bacteriophage, in which the majority of identifiable viruses were Siphophages [10].

Previous studies indicated that the host bacterium, *Bacteroides sp.* GB-124, was most closely related to *B. ovatus* based on comparison of 16S rRNA gene sequences (96% identity; [26]). However, since 97% identity between 16S genes is typically used as the cut off for species level identification [32], and in light of the recent release of many additional *Bacteroides* genome sequences from human gut isolates (as part of the human microbiome project), we investigated the identity of *Bacteroides sp.* GB-124 in more detail. This new analysis revealed GB-124 to be a strain of *B. fragilis* (designated here *B. fragilis* GB-124), with 16S rRNA gene sequences exhibiting 99% identity to a number of other *B. fragilis* strains, including those isolated from the same municipal wastewater site as well as strains HSP40, 683R, YCH46, JCM 17586 and JCM 17587 isolated from various human body sites and human faeces (Table S1, Figure 1B; accession numbers given on figure).

Investigation of the ability of φB124-14 to infect and lyse a range of *Bacteroides spp*. commonly associated with the human gut microbiota, demonstrated that this phage has a highly restricted host range. φB124-14 was capable of infecting only a subset of *B. fragilis* strains isolated from the same municipal wastewater site and *B. fragilis* strain DSM 1396 (Figure 1B; Table S1), originally isolated from human pleural fluid [33]. No activity was observed against other *Bacteroides spp.* tested, or against strains of *B. fragilis* isolated from geographically distinct municipal wastewaters, namely, Galicia, Spain [20] and Hawaii, USA [34] (Figure 1B; Table S1).

Overall, these observations indicate that *Bacteroides spp.* within the human gut microbiota play host to bacteriophage with extremely narrow host ranges, and in at least some cases these are restricted to closely related strains. Such narrow host range may be the result of the extreme niche specialization thought to occur at short phylogenetic distances in gut bacteria [7], likely resulting in strain-to-strain variation in surface proteins or other structures exploited by phage as receptors. In this regard, it is notable that horizontal gene transfer mediated by phage and other mobile elements [35], as well as the selective pressure imposed on host bacteria by phage themselves [6], [36], [37] can all promote modification of surface structures and contribute to strain diversification. In the case of surface structures, since these are often key to host-microbe interactions, and may include those that are important to nutrient acquisition and competition between strains, such diversification also has the potential to influence the interaction of bacteria with the human host [35]. Phage with such restricted host ranges are also unlikely to produce a significant impact on overall microbial community structure and functioning due to functional redundancy among members of the gut microbiome [6], [29].

#### Genome structure and sequence overvi

**Figure 2:**
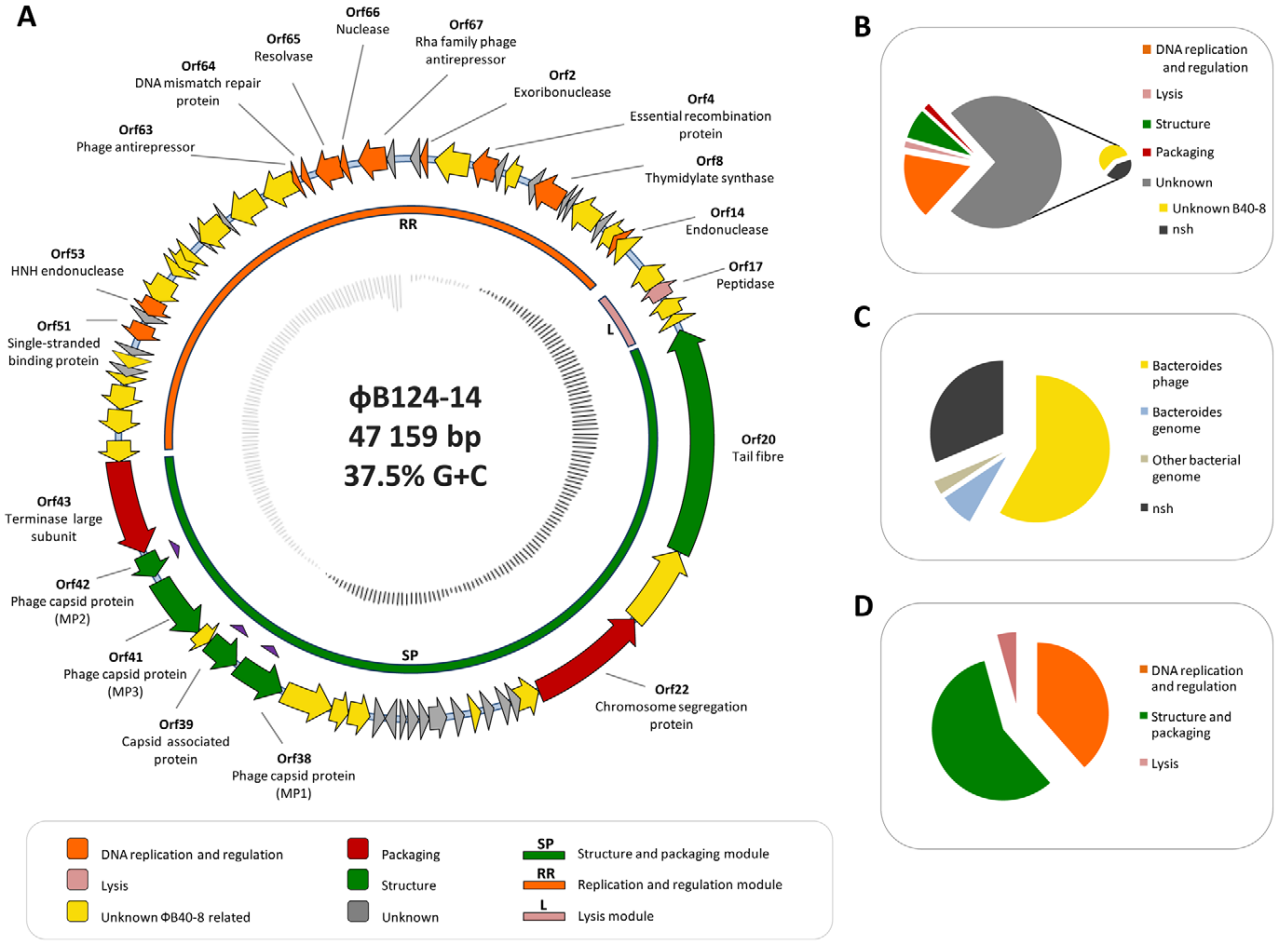
Architecture and characteristics of φB124-14 genome. **A.** Physical map of ΦB124-14 genome. **Outer track:** Position and orientation of each predicted ORF. Block arrows represent individual ORFs and indicate direction of transcription. ORF colour indicates functional assignment based on BlastP and conserved domain searches (minimum 20% identity, and an e-value of 1e^−5^ or lower), as well as analysis of the phage proteome, as described in the figure legend. ORFs marked with purple triangles indicate ORF function has been confirmed through LC-MS/MS analysis of the mature phage proteome (See Figure 4). **Middle track:** Bars represent location of proposed functional gene clusters and ORFs belonging to each cluster. Colours of bars indicate putative role of each gene cluster in phage replication, based on predicted functions of member ORFs. **Inner track:** G+C content: dark grey lines  =  above average genome G+C content; light grey lines  =  below average genome G+C content. **B.** Percentage of ORFs assigned to each functional category, including unassigned ORFs. ORFs of unknown function are further broken down in a secondary pie chart to illustrate those with homologues in the other available *Bacteroides* phage genome (φB40-8), and those with no significant homology (nsh) to any sequences present in public databases encompassed by the nr dataset. **C.** Percentage of φB124-14 ORFs with highest homology (based on top hits by bit score in BlastP searches) to sequences of various phylogenetic origin. Only hits generating e values of 1e^−5^ or lower were considered significant in this analysis. nsh – no significant homology. **D.** Illustrates the percentage of predicted ORFs assigned to each of the three predicted functional modules in the φB124-14 genome.

**Figure 3:**
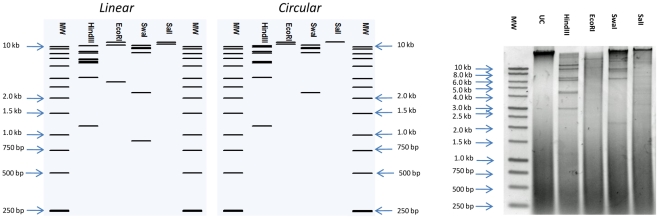
Physical structure of φB124-14 genome. Left and middle panels show *in silico* digest and electrophoresis to visualise restriction fragment profiles of φB124-14 expected for each permutation of the genome (linear or circular) generated by pDRAW32. **Right panel** shows results obtained from digestion of 1.5 µg of φB124-14 DNA (3 h at 37°C) with restriction enzymes used in *in silico* analysis. Restriction enzymes tested are indicated above each lane. MW  = 1 kb Molecular Weight marker (Promega). UC  =  uncut φB124-14 DNA.

The dsDNA genome of φB124-14 is 47,159 bp with an average G+C content of 38.75%, and predicted to encode 68 open reading frames (ORFs) with an average size of 212 aa. The genome exhibits the high coding density typical of bacteriophage, with non-coding sequence limited to 8.2% of the genome (Figure 2A). Restriction fragment patterns are compatible with a circular genome structure, and indicated that the φB124-14 is packaged as a circular molecule (Figure 3), as has been described for bacteriophages P2 and P4 [38], [39].

Similar to the host bacterial species, *B. fragilis*, the majority of putative ORFs detected are predicted to be initiated by an ATG start codon, with one ORF (ORF10) presenting a CTG start codon, and two initiated by GTG codons (ORF34, ORF44). A number of ORF start and stop codons overlap (Table S2; Figure 2A); a feature common to bacteriophage genomes, which has been hypothesised to facilitate gene regulation and allow an increased repertoire of proteins without a corresponding increase in genome size [40], [41]. Based on the protein BLAST algorithm (BlastP; http://blast.ncbi.nlm.nih.gov/Blast.cgi) and proteomic analysis, 18 of the predicted ORFs have an assignable function, and 12 ORFs contain conserved domain signatures (Table S2; Figure 2A). The majority of ORFs with an assigned function encode proteins with predicted roles in DNA replication and regulation, with the remainder predicted to encode functions related to capsid structure, packaging, and host lysis (Table S2; Figure 2A).

No function could be predicted for 50 ORFs which were all designated as proteins of unknown function (Table S2; Figure 2A,B). Of these, 29 were homologous to ORFs within the φB40-8 genome or the genomes of *Bacteroides spp*., and a further 21 exhibited no significant homology to any sequences within the public databases (Table S2; Figure 2A, B, C). This likely reflects the general paucity of bacteriophage genome sequences within public databases (only one other complete *Bacteroides spp*. phage genome [31] is currently available in public databases, as of October 2011), as well as the high level of uncharacterized functions typically encoded by phage genomes [6], [42].

### Genome architecture and phage encoded functions

The clustering of functionally related genes and modular gene architecture is a common feature of bacteriophage genomes. Based on gene architecture, putative transcriptional coupling, and the functional assignments of ORFs, the φB124-14 genome also exhibits a modular organisation with functional gene clusters related to packaging, capsid structure and assembly, as well as DNA replication and regulation, and host lysis (Figure 2A,B; Table S2). A comparable gene architecture and functional clustering has also been described in φB40-8, which exhibits a similar array of loosely defined gene modules containing high levels of ORFs of unknown function [31]. However, many ORFs assigned to particular modules in both φB40-8 and φB124-14 genomes cannot be assigned a specific function due to lack of homology to any sequences in current databases, or any direct experimental evidence.

### Structure and packaging

**Figure 4:**
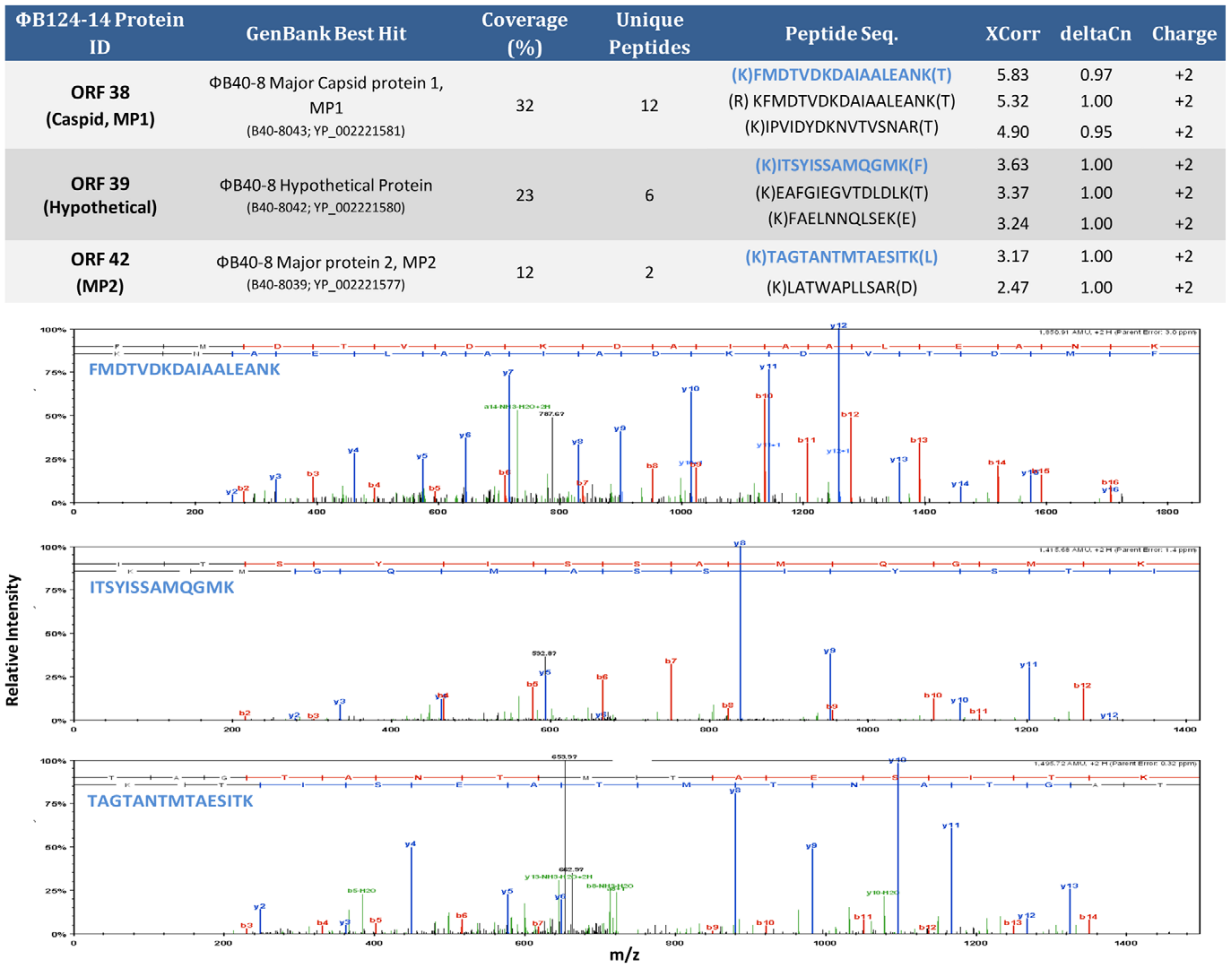
Analysis of the mature φB124-14 proteome. Spectra of φB124-14 proteins identified by tandem mass spectrometry. Example peptide spectra for each of the three proteins identified are shown. Table provides protein coverage and associated number of unique peptides matched and the sequence of the top four matches (ranked by by XCorr score).

The φB124-14 structure and packaging cluster potentially comprises 24 ORFs, constituting 52% of the phage genome (Figure 2A). Across this cluster seven ORFs could be assigned putative functions based on sequence homologies and analysis of the mature virion proteome (Table S2; Figures 2 and 4). ORFs 38, 41 and 42 are predicted to encode the main structural proteins comprising the phage capsid, and exhibit high homology to corresponding capsid proteins from φB40-8 at the amino acid level (MP1 – Major Capsid Protein 1, MP3 – Major Capsid Protein 3, and MP2 – Major Protein 2, respectively; Table S2). ORF20 encodes a putative tail fibre protein, which is thought to be involved in host recognition and phage attachment [43]. However, with the exception of the predicted tail fibre protein, which exhibits homology with other proteins annotated in *Bacteroides spp*. genome sequences, capsid proteins of both phage (φB124-14 ORF38, 39, 41, 42 and corresponding φB40-8 ORFS; Table S2) show no significant homology to any other sequences in BlastP searches of the nr dataset (e = 0.03 or greater), and all lack conserved domains found in other phage capsid proteins.

The presence of ORF38 (φB40-8 Major Capsid Protein 1 homologue), and ORF42 (φB40-8 Major Protein 2 homologue) in the mature virion was confirmed by analysis of the phage proteome by tandem mass spectroscopy (Figure 4). Proteomic analyses also identified an additional protein within the structure and packaging cluster (encoded by ORF39) as present within the mature virion, confirming a role for this previously hypothetical protein in capsid structure (Figures 2 and 4). In contrast, ORF41 and ORF20 (φB40-8 Major Capsid Protein 3 homologue and Tail Fibre homologues, respectively) were not detected in mature φB124-14 phage capsids, and their role in virus capsid structure remains hypothetical.

The lack of identifiable products from ORF41 and ORF20 in the proteome of mature φB124-14 virions may indicate that these are present at relatively low levels that do not permit accurate identification using the proteomic techniques employed here. Although the theoretical sensitivity of the Orbitrap XL LC-MS system utilised in this study implies proteins should be detected at the low fmol range, subsets of comparatively rare proteins can be “masked” by the presence of highly abundant proteins in any given sample [44]. Alternatively, ORF41 and ORF20 may be non-functional in φB124-14 and could be dispensable for capsid formation, or play only transient roles in capsid assembly, potentially limited to aspects that occur *in vivo* in host cells.

In the case of ORF20, the assignment of this as a tail fibre protein is based solely on its similarity to the homologous protein annotated as a tail fibre in the φB40-8 genome sequence. However, a closer examination of both φB124-14 and φB40-8 proteins revealed only low levels of homology to other tail fibre proteins, with the highest homology observed with an *Enterococcus* phage phiEF24C-P2 protein annotated as a tail fibre component (32% identity, 10% query coverage, 6e^−10^). The large size of ORF20 is more typical of tail tape measure proteins, and given the low homology to other tail fibre proteins, and the apparent absence from the phage structural proteome, the possibility that ORF20 encodes a tape measure protein rather than a tail fibre should be noted. Nevertheless, in the absence of experimental evidence demonstrating a specific function, the available genomic data indicates φB124-14 ORF20 to be most closely related to the φB40-8 putative “tail fibre” protein, and the φB124-14 genome has been annotated to reflect this.

#### DNA replication and regulation

ORFs assigned to the DNA replication and regulation cluster account for more than half of all those encoded by φB124-14 but constitute only 35% of the phage genome (Figure 2A,D). The devotion of a large number of ORFs with roles in replication and DNA synthesis is concordant with recent large-scale analyses of the human gut viral metagenome in which genes involved in nucleotide replication and synthesis were found to be enriched [6]. In addition, this is also observed in the only other publically available complete *Bacteroides sp*. phage genome, φB40-8 [31]. However, of the ORFs affiliated to this putative cluster, as with phage encoded ORFs in general, only a small proportion (25%) could be assigned a putative function (Figure 2A, B, C).

Of particular interest in the regulation and replication cluster is a predicted thymidylate synthase (TS; ORF8). TS is a ubiquitous enzyme in bacteria that catalyzes the formation of deoxythymidine 5′-monophosphate (dTMP) from deoxyuridine 5′-monophosphate (dUMP), which is essential for dTTP synthesis and DNA replication [45]. Based on sequence homology and conserved domain searches, the φB124-14 ORF8 appears to encode a ThyA type enzyme which is predicted to be utilised by ∼70% of microorganisms [46], but seemingly rare in human gut viral genomes and most likely acquired from host bacterial species [6]. However, conserved domain searches indicate that the φB124-14 TS may also exhibit dUMP hydroxymethylase activity and thus constitute a bi-functional enzyme involved in the manufacture of modified nucleotides (Table S2). This latter function is thought to protect phage DNA from restriction-based host defence mechanisms [47].

In addition, owing to the importance of TS activity for bacterial survival, it has also been suggested that phage-encoded TS are of benefit to host bacteria [48]. The provision of additional copies of ThyA may enhance bacterial growth through gene dosing effects as well as providing redundancy for a key activity and safeguarding against its loss [48]. Furthermore, the efficiency of thymidylate metabolism has also been implicated as a limiting factor in prokaryote genome expansion and evolution, as well as cell proliferation [46]. Overall, any enhancement in host survival ability and replication rate is also of obvious benefit to bacteriophage, since facilitating survival and replication of host bacteria will contribute directly to phage survival.

The φB124-14 replication and regulation module also encodes recombination proteins (ORF4) and phage antirepressors (ORF63 and 67) (Table S2; Figure 2). Phage encoded recombination proteins are often involved in facilitating recombination between the phage attP site and the attB site in the host chromosome during formation of prophage insertions [49]. Phage antirepressors are also often found in prophage elements [50], and these regulators typically control the activation and de-repression of genes required for re-entry into the lytic life cycle, often in response to changes in the physiological status of the host cells [50].

#### Host lysis

As with the previously characterised φB40-8 [31], φB124-14 lacks a well-defined lytic module, and there is a relative absence of ORFs encoding proteins with an obvious role in host cell lysis. This lack of a well-defined lytic module is also a general feature of other phages belonging to the *Siphoviridae* family [31],[51]. Only one protein (ORF17), encoding a putative M15 type metallopeptidase, could be assigned a clear function potentially related to host lysis; phage-encoded peptidases are often involved in disruption of the host cell envelope [52], [53]. However, ORF17 appears to form part of a small gene cluster with several ORFs of unknown function, which collectively constitute a putative lytic module (Figure 2A). Two of these putative lytic module members (ORFs 16 and 19) are predicted to encompass transmembrane signal sequences and it is possible that these function to target the encoded proteins to the cell wall or periplasm, as is often observed with holin-endolysin systems [54].

### Phage life cycle

Although the φB124-14 genome encodes some genes normally related to temperate life cycles (recombinases, transcriptional repressors and anti-repressors; Figure 2, Table S2), no evidence for a lysogenic cycle was indicated in previous host range analyses. The existence of φB124-14, or homologous elements, as prophage was investigated within currently available *Bacteroides* genome sequences using the nucleotide BLAST algorithm (Blastn). Although lytic replication of φB124-14 appears to be confined to only a few closely related strains of *B. fragilis* (Figure 1B), this investigation encompassed 48 available complete and draft genomes (Table S3), within the genus *Bacteroides*, including human gut-specific species. A broad range of *Bacteroides* species was included in this analysis to account for the possibility that an alternate life cycle may occur in species other than *B. fragilis*, which may not be detected under the laboratory conditions used to elucidate host range in this study.

However, no evidence for a lysogenic life cycle or the existence of φB124-14 as prophage was provided by this analysis, with all chromosome sequences analysed devoid of any detectable φB124-14-like prophage regions. Moreover, in addition to the production of clear plaques and the absence of any identifiable integrase genes, the large deviation between G+C content of the potential bacterial host *B. fragilis* (G+C∼43.3%) and φB124-14 (G+C 37.5%) is also fitting with a lytic rather than lysogenic lifestyle [55]. Although deviation in G+C content may also be evident in lysogenic prophage, a propensity for a larger reduction in the G+C content of lytic phage, as well as the resulting increases in genome signature differences (in terms of nucleotide repeat patterns), have been used as indicators of a lytic lifestyle [55], [56]. Thus, despite the presence of genes often associated with a lysogenic cycle in other phage, there is currently no evidence to indicate φB124-14 undergoes a temperate life cycle. Considering also the dynamic and mosaic nature of phage genomes, in the case of φB124-14 genes such as anti-repressors could conceivably be remnants of previous genomic incarnations, which no longer undertake their original function.

### Comparative genome analysis of available Bacteroides phage sequences

Annotation and analysis of the φB124-14 genome sequence indicated many ORFs were homologous to predicted proteins from *Bacteroides* φB40-8 [31], also a member of the *Siphoviridae* family and originally isolated from an urban sewage sample [57]. In order to examine the similarity between both phage in detail, a comparative genomic analysis of the φB124-14 and φB40-8 complete genome sequences was undertaken.

**Figure 5:**
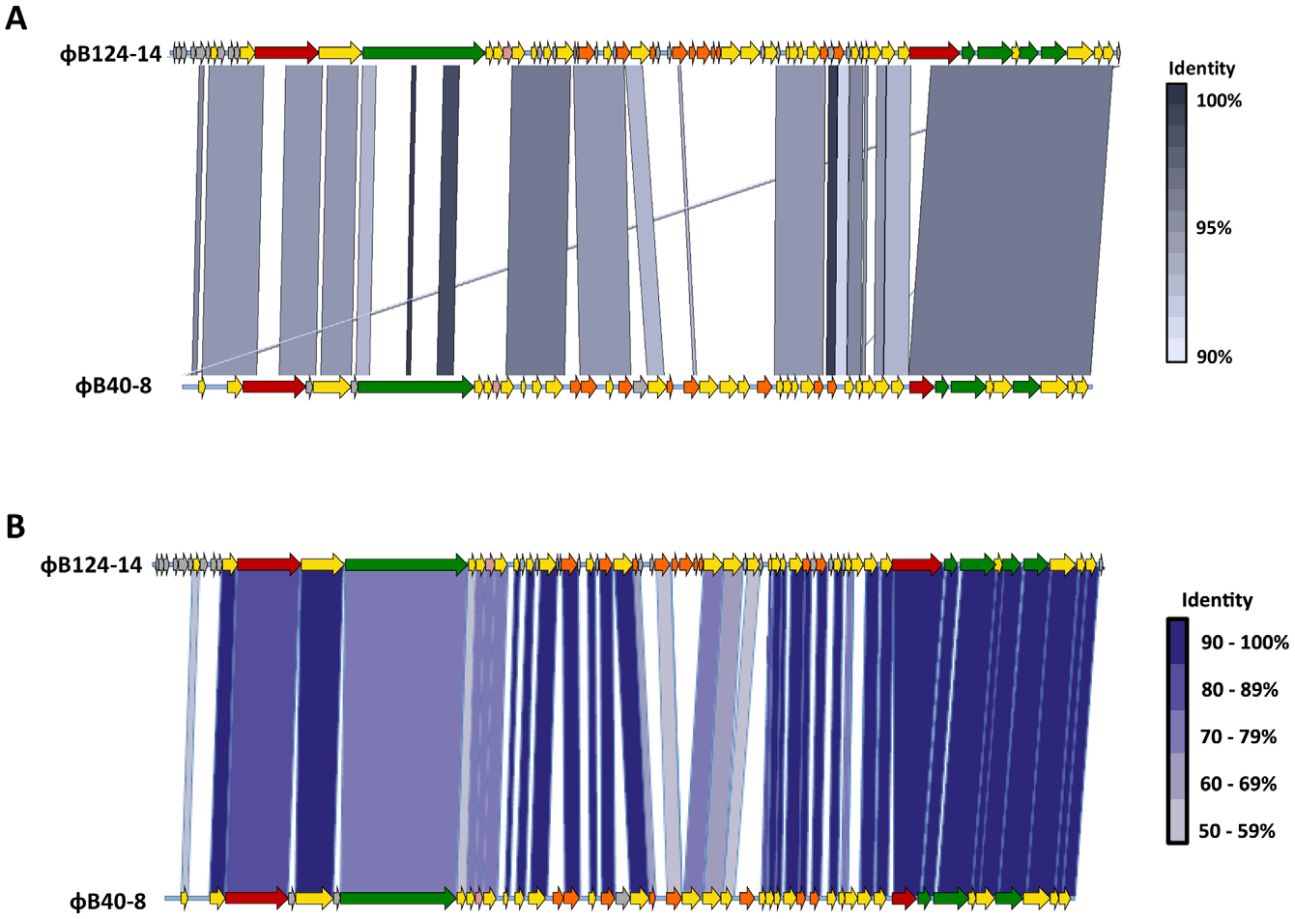
Comparative genomic analysis of ΦB124-14 and ΦB40-8 (ATCC 51477-B1). **A.** Nucleotide sequences of φB124-14 and φB40-8 were compared using the Artemis Comparison Tool (ACT). Shaded areas between linear phage genome maps represent areas of high nucleotide identity (90% or greater). Colour scale represents level of nucleotide identity at each region of homology. The ORF map for φB40-8 corresponds to the annotations available in the GenBank submission (FJ008913.1). For the purposes of this analysis, the φB124-14 genome was linearised between ORFs 29 and 30 (Figure 2, Table S2), in order to compare the circular φB124-14 genome with that of φB40-8. Colours of ORFs correspond to functional assignments as used in Figure 2. **B.** Comparison of amino acid sequences from φB124-14 ORFs with those annotated in the φB40-8 genome. Shading between arrows indicates those sharing high amino acid sequence identity. Colour scale indicates level of amino acid identity between each homologous ORF.

Direct comparison of φB124-14 and φB40-8 complete genome nucleotide sequences using the Artemis Comparison Tool (ACT; [58]) (Figure 5A), as well as ORF-by-ORF comparison of translated amino acid sequences (Figure 5B) revealed significant homology over large areas of the phage genomes, encompassing regions believed to be involved in structure and packaging, DNA replication and regulation, and lysis, with a general conservation in gene architecture and organisation evident (Figure 5). At the nucleotide level, φB124-14 and φB40-8 are 57% identical across the complete genome sequences, with the majority of ORFs in each genome exhibiting homologues in the other (Figure 5). Concordantly, Coregenes [59] analysis, which determines the core set of genes common to two or more distinct genomes, showed that 39 of φB124-14 ORFs are shared with φB40-8 (BlastP identity of 75% or over), with structural genes displaying particularly high levels of homology (96% identity or greater, Figure 5B).

### Comparative metagenomic analysis of φB124-14 and φB40-8

Due to the absence of phage infecting the host strain (*B. fragilis* GB-124) from faecal samples derived from a wide range of common domestic and wild animals, and from the general environment, our previous work strongly suggested φB124-14 is human gut specific [20]. φB40-8 has also been utilised as a marker of human faecal pollution and is thought to be indicative of the human gut microbiota [57]. To provide further insight into the distribution of φB124-14 and φB40-8 in various microbiomes, and evaluate their utility for the development of culture-independent faecal source tracking methods, we undertook a comparative metagenomic analysis using both complete bacteriophage genome sequences.

The general distribution of sequences with homology to φB124-14 and φB40-8 was investigated within all publically available metagenomic datasets in the NCBI metagenome database (as of June 2011, excluding those comprised solely of 16S rRNA gene sequences), as well as the microbiomes of 124 individuals of European origin which comprise the METAHIT dataset [28], 2 individuals of American origin [60], 13 Japanese individuals [8] and the viromes of 12 individuals of American descent [6].

Searches using the full length phage nucleotide sequences failed to identify metagenomic sequences with significant homology (defined as a minimum of 80% identity over 100 nucleotides or more, with an e-value of 1e^−5^ or lower) to either phage in any of the available non-human gut metagenomes searched, or within the available environmental metagenomes of aquatic and terrestrial origin. Interestingly, sequences with high homology to φB124-14 and φB40-8 were detected within the termite gut metagenome [61], but these were below the 80% identity threshold considered to be significant for the purposes of this survey (≤71% identity in the termite metagenome). However, it should be noted that no dataset currently provides complete coverage of representative microbial communities and associated MGE.

The lack of homology to both phage within non-human gut metagenomes will almost certainly reflect the distribution of bacterial hosts in various microbial habitats. In the case of φB124-14, the narrow host range observed for this phage supports previous findings that the *B. fragilis* host strains it infects (Figure 1B) are specific to the human gut [20].

**Figure 6:**
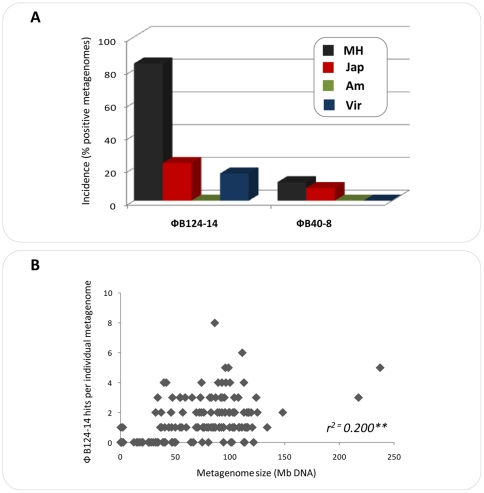
Incidence of sequences homologous to ΦB124-14 and ΦB40-8 human gut metagenomes. Percentage of individual metagenomes in which sequences homologous to φB124-14 or φB40-8 were identified (≥80% identity over ≥100 nucleotides, 1e^−5^ or lower). The microbial metagenomes examined were derived from individuals of European (MetaHit) [28], Japanese [8] and American [60] origin, alongside the combined viromes from 12 individuals of American descent [6]. **MH MetaHit**– All individuals represented in the MetaHit dataset; **Jap** – All individuals of Japanese origin; **AM** – All individuals of American descent; **Virome** – All viromes from individuals of American origin. **B.** Scatter plots illustrating the relationship between size of individual metagenomes searched and detection of sequences homologous to φB124-14. r^2^ =  Pearson correlation co-efficient. **P<0.0001.

Concordantly, sequences with homology to φB124-14 were present in 104 of 124 (83.8%) human gut metagenomes within the MetaHIT dataset (comprised of Danish and Spanish individuals), 3 out of 13 (23%) Japanese individuals and 2 out of 12 human gut viromes (16.6%) (Figure 6A). By contrast, homologous sequences to φB40-8 were detected in only 11.1% of individual MetaHIT metagenomes, in only one gut metagenome of Japanese origin and no homologous sequences were found in the 12 human gut viromes searched (Figure 6A). Importantly, the observed incidence of sequences homologous to both phage was only very weakly positively correlated to the size of metagenomes (r^2^ = 0.2; *P*<0.0001; Figure 6B), indicating that observed differences in incidence are unlikely to be an artifact of differing metagenome size.

In contrast, homologous nucleotide sequences to both phages were absent from the gut metagenomes of the two American individuals [60] examined, with the search criteria employed. This is perhaps unsurprising given the lack of sequences from *Bacteroides spp*. within the metagenomic datasets generated by Gill and co-workers [60]. However, a lack of homology to both phage was also apparent in the American gut viral metagenomic dataset available on GenBank at the time of study [62]. Given that this dataset focuses on RNA viruses [62], a lack of homology to the DNA viruses φB124-14 and φB40-80, was also not unexpected. However, a general lack of homologous sequences was also apparent in the gut viral metagenomes of American origin generated by Reyes and colleagues (Figure 6A) [6].

Despite these caveats, the current observations indicate potential geographic variation in the distribution of these phage, and may also reflect inter-individual variation in actual levels of bacteriophage resident in distinct human gut microbiomes. This notion is congruent with observed differences in excretion of phage amongst the human population; with *Bacteroides* HSP40 infecting phages such as φB40-8 shown to be excreted by a lower number of individuals than other *B. fragilis* phages [23], [63]. These differences are likely also indicative of the abundance of host strains within the human gastrointestinal tract (GIT), as well as intra-individual differences in gut viral community population structure.

### φB124-14 ecological profiling

To further evaluate the potential utility of φB124-14 in broad-scale MST applications, and the putative gut specific nature of this phage (as indicated by previous studies [20], [26], [34] and our comparative metagenomic analysis) the relationship of φB124-14 with the wider bacteriophage community was explored. This was investigated using both conventional gene-centric alignment-driven phylogenetic analysis, as well as gene-independent alignment-free methodologies based on the pattern of tetranucleotide repeat frequencies encoded in the φB124-14 genome [51], [64]–[65]. In particular, the latter approach facilitates large-scale analyses of nucleotide sequence affiliation and relationships, which permit a more expansive overview of φB124-14 ecology.

#### φB124-14 terminase based phylogeny

**Figure 7:**
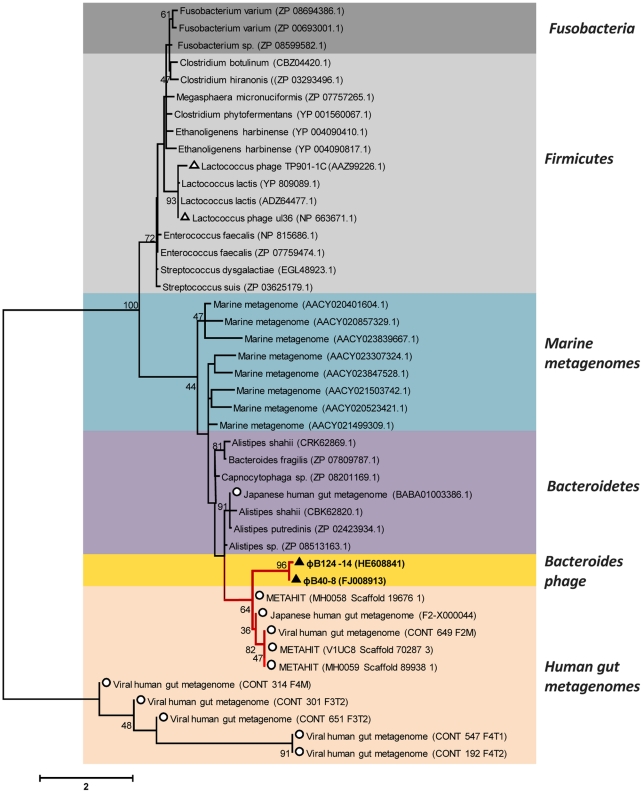
Phylogeny of ΦB124-14 large subunit terminase. Amino acid sequences homologous to φB124-14 terminase (ORF43), based on bit-score, were retrieved from GenBank and metagenomic datasets, including human gut microbiomes and viromes [6], [8], [28] and marine microbial metagenomes [92], and aligned using ClustalW. The unrooted concensus neighbour joining tree (1000 bootstrap resamplings) was produced using MEGA v5. Bootstrap values ≥40 are shown adjacent to respective tree nodes. Scale indicates amino acid substitutions. Colours indicate phylum level grouping or origin of metagenomic sequences. Black triangles indicate φB124-14 or φB40-8 terminase sequences; white triangles represent other phage sequences; white circles represent sequences originating from human gut metagenomes.

Since terminases are thought to be the most highly conserved gene within phage [66], conventional phylogenetic analysis was undertaken using the putative φB124-14 terminase gene (ORF43) (Figure 7). Homologous amino acid sequences from phage and bacterial genomes (prophage), as well as from metagenomes of diverse origin, were aligned with the predicted large subunit terminase of φB124-14, and alignments used to construct phylogenetic trees. This analysis further confirmed the close association of φB124-14 with φB40-8, and also revealed a strong association with predicted terminase and terminase-like sequences originating from human gut microbiomes [8], [28] and viromes [6].

As expected, the closest association was observed with terminase sequences originating from human gut-associated members of the Bacteroidetes division, including *B. fragilis* (the host species of φB124-14), and *Alistipes spp*. (Figure 7). In addition, the majority of terminase sequences derived from human gut viral metagenomes [6] represented in this tree appeared to be distinct from all other sequences retrieved from other sources (Figure 7). This latter observation suggests the existence of additional gut-specific bacteriophage and hints at a close association between the human host, its microbiome and components of the associated mobile metagenome. However, this phylogenetic analysis was limited to sequences possessing terminase genes closely related to that of φB124-14, and also to those generating good alignments with the φB124-14 sequence. By default this excludes the majority of metagenomic virome sequences (due to the fragmentary nature of such datasets), and provides only a limited view of φB124-14 ecology and evolution.

#### Gene-independent genome signature-based ecological profiling

In light of the narrow view offered by gene-centric alignment-based phylogenetic methods for analysis of φB124-14, and the problems associated with expanding such surveys when analyzing bacteriophage genomes in general, we next explored the broader ecological landscape occupied by φB124-14 using gene-independent and alignment-free methods [51], [64], [65].

Since bacteriophage and other mobile genetic elements are believed to reflect the genomic signatures of their host bacteria (in terms of di-, tri-, and tetra-nucleotide repeat frequency (TRF) patterns; [51], [67]), it would be expected that bacteriophage with similar host ranges will exhibit comparable TRF signatures. Therefore, comparison of these TRF genetic signatures may be used to place φB124-14 in a wider ecological context with other bacteriophage, bacterial host species, and sequences obtained from metagenomic datasets.

**Figure 8:**
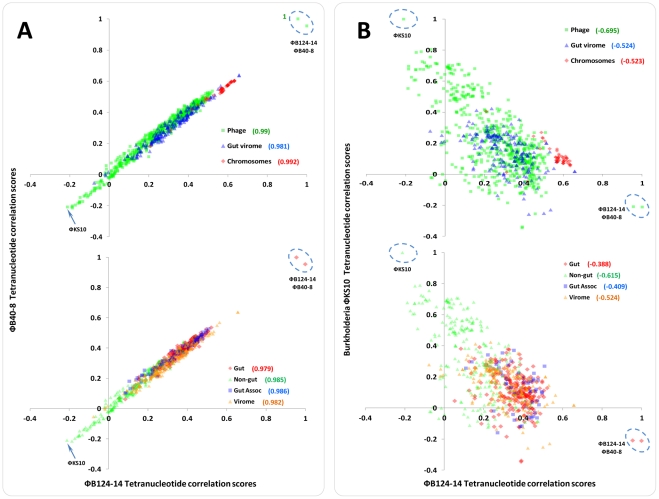
Comparison of tetranucleotide repeat frequency patterns in bacteriophage genomes and ecological profiling of φB124-14 and φB40-8. The tetranucleotide repeat frequency (TRF) correlation scores for φB124-14, φB40-8 and *Burkholderia* φKS10, were compared using scatter plots and correlation of data examined using the Pearson coefficient. A complete list of genomes and sequences utilised in this analysis is provided in Table S3. **A.** Comparison of TRF scores for φB124-14 (x-axis) vs φB40-8 (y-axis). **B.** Comparison of TRF scores for φB124-14 (x-axis) vs *Burkholderia* φKS10 (y-axis). **A, B. Upper charts** plot scores for all phage genomes, viral metagenome fragments, and *Bacteroides* genomes. **Phage**  =  TRF scores from comparisons to 611 phage and prophage genomes. **Virome**  =  TRF scores from comparisons to 188 large fragments (>10 Kb) from human gut viral metagenomes [6]. **Chromosome**  =  TRF scores from comparison to 48 *Bacteroides spp*. genome sequences. Each sequence type is represented by a different colour and symbol as indicated in the figure legends on each chart. The intensity of shading of data points reflects the number of data points represented in a given area with a greater intensity indicating more overlapping data points. Values in parentheses provide Pearson correlation scores for each sequence type. **Lower charts** plot TRF scores for sequences assigned to one of three categories based on their relation to the human gut microbiome: **Gut** **= ** comprises bacteriophage infecting bacterial genera commonly forming part of the normal human gut microbiota. **Gut Associated  = ** comprises bacteriophage genomes infecting bacterial genera whose member species are associated with the gut but not generally considered to be members of the normal gut microbiota (such as primary invasive gut pathogens), or where member species are more commonly associated with environmental habitats. **Non-Gut  = ** contains bacteriophage infecting bacterial genera with member species not considered to be part of the human gut microbiota or typically associated with this community, and primarily encompasses bacteriophage infecting genera of environmental origin. **Virome  = ** All large fragments (n = 188, >10 Kb) assembled using CAMERA workflows (per individual) from human gut viral metagenomic libraries [6], [91]. Each sequence category is represented by a different colour and symbol as indicated in the figure legends on each chart. For the purposes of this analysis phage infecting a particular host bacterial genus were only utilised if four or more representative phage genomes were available (540 complete phage genomes, representing 31 bacterial genera). The intensity of shading of data points reflects the number of data points represented in a given area with a greater intensity indicating more overlapping data points. Values in parentheses provide Pearson correlation scores for each sequence type.

To this end we compared the patterns of TRF in the genome of φB124-14 to those encoded in the genomes of 611 other bacteriophage, 48 chromosomal sequences from a range of *Bacteroides* species, and all large fragments (>10 kb, n = 188 contigs) assembled from human gut meta-viromes generated by Reyes *et al.* [6]. In light of the similarities observed between φB124-14 and φB40-8 in other analyses undertaken here, TRF scores for each bacteriophage were correlated to identify ecological similarities or differences. This not only permitted the evaluation of the effectiveness of this genetic signature-based approach but also the exploration of the extent to which the ecological landscapes populated by both phage overlap (Figure 8).

In general, results of this analysis were congruent with host range studies, comparative genomic analyses, and trends observed from construction of terminase phylogenetic trees (Figures 1, 5 and 7). As expected, φB40-8 was the most closely related bacteriophage to φB124-14 and sequences from *B. fragilis* strains were found to be the most closely related chromosomal sequences (Figure 8A; Figure S1). A high level of correlation was observed between TRF scores derived from the comparison of each phage (φB124-14 and φB40-8) against all other sequences analyzed (r = 0.981 or above; Figure 8). This high level of correlation indicates that both phage share closely related and highly similar ecological niches, in keeping with known host ranges, and the close phylogeny and evolutionary relationship observed in our other investigations (Figure 8A).

The relationship of other complete bacteriophage genomes (relative to φB124-14 and φB40-8) also exhibited a marked trend based on the broad classification of bacterial host genera and its association with the human gut microbiome (Figure 8A). The genomes of phage infecting bacterial genera commonly found in the human gut microbiome displayed a clear association relative to the φB124-14 and φB40-8 genomes, forming a distinct grouping centered around the majority of gut virome sequences (Figure 8A).

In contrast, those exhibiting the least similar TRF profiles were phage infecting bacterial genera predominantly associated with terrestrial, aquatic or marine environments and not members of the normal human gut microbiota (Figure 8A). Identical analyses utilizing the φB124-14 genome, and that of *Burkholderia* φKS10 [68] (which generated the lowest TRF correlation to φB124-14 of all phage analyzed; Figure 8A) displayed none of the trends observed between φB124-14 and φB40-8, and exhibited only negative correlation coefficients (r = −0.409 or below) with φB124-14 in relation to sequence categories or groups used (Figure 8B). However, even in this analysis, gut-associated phage genomes, bacterial genomes, and gut virome fragments were observed to be much more closely affiliated with φB124-14, displaying a distinct trend towards the φB124-14 axis (Figure 8B). Collectively, these observations confirm the usefulness of the TRF approach to investigate bacteriophage ecology (Figure 8).

Despite the observed trends and phage groupings, much overlap was observed between bacteriophage assigned to different categories, an observation that is not unexpected in light of the constant ingress of “contaminants” into the gut ecosystem through consumption of food, the malleable nature of phage genomes, and the broad categories to which phage genomes were assigned in this analysis. Nevertheless, the relationships indicated here suggest that φB124-14 and φB40-8 have a strong association with the gut microbiota and occupy a distinct and largely uncharacterized ecological niche in this community.

As well as facilitating the development of novel MST tools, genomic characterization of phage infecting prominent members of the human gut community also provides fundamental insight into a fraction of the mobile metagenome that constitutes an immense and largely unexplored gene-space. This fraction of the gut microbiome is likely to encode novel activities relevant to development and functioning of the human GIT, and be of pharmaceutical or biotechnological interest in its own right [1], [9]. This is particularly relevant for phages infecting members of the Bacteroidetes which constitute a major component of the human GIT microbial community [28], and have been implicated in both the onset of and protection against the development of gut-related disorders [69]–[71]. Given the potential for phage to shape microbial community structure and function [10]–[15], coupled with their highly selective nature, the isolation and characterization of gut-specific phage offer numerous possibilities for the therapeutic manipulation of the human gut microbiota, and a range of biotechnological applications including the development of novel MST tools.

In this regard the genetic characterization of φB124-14 has provided an essential first step in the development of culture-independent microbial source tracking tools. In particular PCR-based tools that will permit sensitive detection and quantification of human gut-specific indicators (such as φB124-14 DNA), will be made possible by the availability of this, and other, genome sequences of human gut-specific bacteriophage. In this regard, current efforts in developing portable, self-contained “chip” style PCR systems, for accurate and rapid diagnosis of bacterial infections at point-of-care [72]–[73], will translate well for microbial source tracking applications. Ultimately, such methods will eliminate the need for anaerobic culture, permitting rapid and sensitive monitoring of faecal pollution in a range of samples from surface water to shellfish.

Our analyses have also provided insight into a novel and uncharted ecological landscape within the human gut microbiome. Comparative metagenomic analysis, along with ecological profiling confirmed the gut-specific nature of φB124-14, corroborating our previous findings [20]. Intriguingly, this analysis also indicated that φB124-14 and φB40-8 genomes are distinct from other phage genomes and the meta-virome sequences examined here, seemingly occupying an ecological sphere of the human gut virome not represented in currently available human gut meta-viromes, and by only two phage genomes (φB124-14 and φB40-8) in public sequence databases.

In conjunction with the apparent broad geographical distribution of sequences homologous to φB124-14 in human gut microbiomes (observed in our comparative metagenomic analysis), this observation points to a long-term association with the human gut microbiome. In keeping with this hypothesis is the observation that both φB124-14 and φB40-8 encode functions (namely TS) previously found to be absent from extensive viral datasets, but present in gut-associated viral metagenomes [6], and which are likely to play a role in wider metabolism and fitness of bacterial hosts. If so, such phage may also contribute to more subtle mechanisms influencing community structure and help shape this ecosystem not only through selective elimination of host species, but also through effects on host fitness and inter-strain or inter-species competition [74]–[76].

However, the relative lack of homologous sequences to these phage observed in comparative metagenomic analysis of American datasets, suggests that phage complements may vary between geographically distinct populations; for source tracking applications region or population specific phage may be required, a picture that is also emerging from other studies [20], [26], [34]. In addition, the large degree of inter-individual variation in the human gut microbiome almost certainly extends to the mobile metagenome, including the virome [6]. In this regard the goal of developing a truly universal MST will most likely require the utilization of multiple gut-specific elements, such as bacteriophage, to construct a multivalent tool capable of detecting a range of human faecal indicators.

Although much of the bacteriophage genetic landscape is exceedingly poorly characterized in the majority of microbial ecosystems investigated to date, including the human gut, here we provide a glimpse of this biological dark matter and its corresponding ecological context. Our findings suggest that the gene-space and ecological neighborhood populated by φB124-14 and related *Bacteroides* phage is even less well characterized than other aspects of the gut virome, and may be almost entirely uncharted at present. The availability of the complete genome sequence of this and other such phage will now permit further study of this aspect of the human gut mobile metagenome, facilitate interpretation of metagenomic datasets, as well as the development and application of novel, sensitive, and rapid culture-independent MST tools.

## Materials and Methods

### Phage, host strains and growth conditions

φB124-14 was originally isolated from municipal wastewater and is routinely propagated on *Bacteroides* sp. GB-124, as described previously [20]. Phages were isolated by the double-agar protocol (ISO 10705-4) [77] developed specifically for *Bacteroides* phages using *Bacteroides* phage recovery medium (BPRM, per litre: meat peptone, 10 g; casein peptone, 10 g; yeast extract, 2 g; NaCl, 5 g; monohydrated l-cystein, 0.5 g; glucose, 1.8 g; MgSO_4_.7H_2_O, 0.12 g; CaCl_2_ solution (0.05 g/ml), 1 ml; hemin, 10 ml of a 0.1% (w/v) solution made up in NaOH 0.02%; 1M Na_2_CO_3_, 25 ml; pH 6.8±0.5).

To ensure purity of φB124-14 isolates, agar plugs containing single φB124-14 plaques (zones of lysis) were picked from plates using a sterile Pasteur pipette and incubated at 4°C for 4 h in 400 µl phage isolation buffer (19.5 mM Na_2_HPO_4_, 22 mM KH_2_PO_4_, 85.5 mM NaCl, 1 mM MgSO_4_, 0.1 mM CaCl_2_), and phage presence was retested using the double-agar method above to generate fresh plaques. This process was repeated three times and the final purified phage suspension used to generate high titre phage stocks for sequencing and other assays.

To generate high titre phage stocks, pure φB124-14 phage suspensions were added to 27 ml of an exponential *Bacteroides* sp. GB-124 culture (OD_620_ 0.33; cell density of approximately 2×10^8^ colony forming units; CFU) and incubated anaerobically (according to [77]) overnight at 37°C to produce crude lysates. Phage lysates producing plaques were subsequently added to 620 ml of GB-124 (OD_620_ 0.33) and incubated overnight as before. Phage suspensions were then purified and concentrated using polyethylene glycol 8000 [78] as follows: NaCl was added to a final concentration of 1 M and phage suspensions were incubated for 2 h at 4°C, then centrifuged at 1800×*g* for 10 min to remove bacterial debris. Polyethylene glycol 8000 was added to a final concentration of 10% (w/v), mixed for 30 min, and left overnight at 4°C. Precipitated phage were collected by centrifuging at 11,000×*g* for 10 min at 4°C. Resulting supernatant was discarded and 30 ml of phage isolation buffer (as above) was added. Suspensions were stored at 4°C overnight, mixed gently to dissolve pellet and centrifuged at 1500×g for 10 min to remove remaining debris. Phage suspensions were filtered through a 0.2 µM polyvinylidene filter (Sartorius, UK). High titre stock suspensions of 10^11^ plaque forming units (pfu)/ml were stored in glass vials in the dark at 4°C.

### Transmission Electron Microscopy (TEM)

Purified phage particles (10^9^ pfu/ml) were immobilised on a 200 mesh Formvar/Carbon copper electron microscope grids (Agar Scientific, UK), and negatively stained with 1% uranyl acetate. Phage were imaged by TEM using an Hitachi-7100 TEM at 100 kV. Phage dimensions were estimated from positively stained micrographs and values reported are the mean value ± standard deviation (SD) of five virion measurements.

### Analysis of φB124-14 host range

Purified phage particles (10^3^ pfu/ml) were tested for their ability to infect and replicate within a selection of host strains using the double agar method as previously described [77]. Plates were incubated for 24 h at 37°C, under anaerobic conditions and presence of plaques was used to indicate ability to replicate in a particular *Bacteroides* species. A number of strains previously isolated from municipal wastewaters from a variety of geographical locations [26] as well as typed *Bacteroides spp*. were tested (see Table S1 for full list of strains and species used). Novel strains were identified further by 16S rRNA gene sequencing, from 16S PCR products amplified with universal primers 27f and 1492r [79] using standard conditions. Purified PCR amplicons were sequenced directly by GATC Biotech AG (Konstanz, Germany) using Sanger sequencing, and are deposited in the EMBL database under the following accession numbers: HE608156, HE608157, HE608158, HE608159 and HE608160.

### *Bacteroides* host species phylogeny

The relationship between the φB124-14 *Bacteroides fragilis* host strain GB-124 and other *Bacteroides* species was examined in closer detail by construction of phylogenetic trees based on 16S rRNA gene sequences. In addition to those 16S sequences generated in this study, sequences homologous to the φB124-14 host species 16S rRNA were retrieved from GenBank based on best-hit Blast analysis and aligned using ClustalW [80]. Evolutionary histories were inferred by constructing consensus maximum likelihood phylogenetic trees based on the Tamura-Nei model using MEGA v5 [81]. The reliability of tree nodes was evaluated using %-age of 1,000 bootstrap resamplings, with bootstrap values ≥40% used to define well-supported clusters of 16S rRNA gene sequences.

### DNA extraction and sequencing

DNA was extracted from high titre phage stocks (10^10^ pfu/ml), as described previously [82], with minor modifications. Briefly, each ml of phage stock was treated with DNAseI (1 µg/ml) and RNAseA (100 µg/ml) to remove contaminating bacterial DNA, before precipitating with 2M ZnCl_2_ (20 µl/ml) for 5 min at 37°C. Precipitate was centrifuged (1 min, 5,000×g) and resultant supernatant discarded. The remaining pellet was gently resuspended in TES buffer (0.1 M Tris-HCl, pH 8; 0.1 M EDTA; 0.3% SDS) and incubated at 60°C for 15 min. Proteins and polysaccharides were precipitated using 3 M potassium acetate (pH 5.2) on ice for 15 min, then centrifuged for 1 min at 8,000×g. DNA in the resultant supernatant was precipitated with isopropanol and centrifuged. The resulting DNA pellet was washed with 70% ethanol, air dried at room temperature and resuspended in 20 µl Tris-EDTA buffer (10 mM Tris-HCl, pH 8; 1 mM EDTA). The complete genome sequence of φB124-14 was obtained by pyrosequencing using a Roche GS FLX with Titanium chemistry. A total of 16,952 reads with an average length of 355 nt were generated and assembled using the GS De Novo Assembler. The final assembly provided average sequence coverage of ∼127× for the φB124-14 genome. All sequencing and genome assembly was conducted by GATC Biotech AG (Konstanz, Germany). Genome size was confirmed by restriction digest and agarose gel electrophoresis, and fragment sizes calculated using Gene Tools software (Syngene, UK). The complete φB124-14 genome has been deposited in the EMBL database under the following accession number: HE608841.

### Annotation and bioinformatic analyses of φB124-14 genome

Open reading frames (ORFs) encoded by φB124-14 were predicted using Glimmer (v3) [83], and annotated using Artemis [84]. The putative function of predicted ORFs were assigned based on homologies to proteins and protein conserved domains identified in BlastP and tBlastn [85] searches against the NCBI-nr, and Conserved Domains Database (CDD; encompassing all NCBI entries plus protein models from Pfam, SMART, COG, PRK and TIGRFAM, and ACLAME databases), respectively.

For BlastP and tBlastn searches only homologous sequences generating e-values of lower than 1e^−5^ at ≥20% identity were considered significant. For Conserved Domain searches, only hits with an e-value of 0.01 or lower were considered significant. Putative tRNA-encoding genes were searched for using tRNAscan-SE [86]. Transmembrane proteins and signal peptides were predicted using the TMHMM v2 [87] and SignalP v3 [88] servers. The presence of prophage with homology to φB124-14 and φB40-8 in complete bacterial genome sequences were predicted using Prophinder [89] and Blastn analysis of *Bacteroides* genomes available within GenBank (See Table S3 for list of genomes). Comparative analysis of bacteriophage genomes was carried out using the Artemis Comparison Tool (ACT) [58]. Physical maps of the annotated φB124-14 and φB40-8 genomes were generated using Vector NTI Advance (v11.5).

### Physical structure of phage genome

Phage genomic DNA was digested with HindIII, EcoRI (Promega. UK), SwaI and SaII (NEB, UK), respectively, for 3 h at 37°C and fragments resolved on a 0.8% Tris Acetate EDTA (TAE) gel at 80 V for 3 h. Resulting restriction fragment profiles were compared to *in silico* restriction profiles for linear or circular permutations of the genome, which were generated by pDRAW32 (http://www.acaclone.com/).

### Analysis of the φB124-14 proteome

φB124-14 lysate (10^11^ pfu/ml) was filtered through a sterile 0.2 µM low protein binding filter (HT Tuffryn, Pall Corp.) to remove cell debris. Resulting crude protein extract was diluted with an equal volume of 2,2,2-Trifluoroethanol (Fluka), 20 mM DTT, and denatured and reduced at 60°C for 60 min, before alkylation with 30 mM IAA at room temperature in the dark for 45 min. The sample was diluted 6-fold with 50 mM ammonium bicarbonate and digested with sequencing grade trypsin (Promega, UK) overnight at 37°C. Tryptic peptides were fractionated on a 250 mm ×0.075 mm reverse phase column (Acclaim PepMap100, C18, Dionex) using an Ultimate U3000 nano-LC system (Dionex) and a 2 h linear gradient from 95% solvent A (0.1 % formic acid in water) and 5% B (0.1% formic acid in 95% acetonitrile) to 50% B at a flow rate of 250 nL/min. Eluting peptides were directly analysed by tandem mass spectrometry using a LTQ Orbitrap XL hybrid FTMS (ThermoScientific) and derived MS/MS data searched against φB124-14 amino acid sequences using Sequest version SRF v. 5 as implemented in Bioworks v 3.3.1 (Thermo Fisher Scientific), assuming carboxyamidomethylation (Cys), deamidation (Asn and Gln) and oxidation (Met) as variable modifications. Filtering criteria used for positive protein identifications are Xcorr values greater than 1.9 for +1 spectra, 2.2 for +2 spectra and 3.75 for +3 spectra and a delta correlation (DCn) cut-off of 0.1.

### Comparative metagenomic analysis

Comparative metagenomic analysis were conducted as previously described [1], [4], [90]. The presence of φB124-14 and φB40-8-like sequences among available metagenomes was investigated in the first instance using the full set of microbial metagenomes of diverse origin available within the NCBI database (158 metagenomes, June 2011). A more detailed investigation of the distribution of φB124-14 and φB40-8-like sequences within the 124 human gut microbial metagenomes from individuals of European descent represented in the METAHIT dataset [28], 13 individuals of Japanese origin [8], 2 individuals of American origin [60] and within the viral metagenomes from 12 individuals of American descent [6] was then carried out. To obtain assemblies of viral gut metagenomes for these analyses, pyrosequencing reads for project SRA012183 [6] were obtained from the NCBI Short Read Archive and processed using CAMERA workflows [91]. Reads were filtered to remove low quality reads and duplicates using the 454 QC and 454 Duplicate Clustering workflows, respectively, with default parameters. The resulting high-quality, non-redundant data sets were assembled using the CAMERA Meta-Assembler which combines output from seven independent short read assemblers run using pre-optimised parameters: Newbler, Taipan, Celera, Velvet, SOAPdenovo, ABySS and SSAKE [91]. Individual metagenomes were processed separately. The combined metagenomes from each dataset (MetaHIT, Japanese gut, American gut and gut viral) were searched using Blastn for nucleotide sequences with homology to φB124-14 and φB40-8. Only sequences exhibiting an identity of 80% or greater over 100 bp or longer at 1e^−5^ or lower were considered significant and used to calculate incidence of positive metagenomes as described previously [1], . Correlation analysis (Scatter plots and Pearson correlation co-efficient) was carried out using Microsoft Excel.

### Ecological profiling of φB124-14

Alignment-driven phylogenetics was undertaken using the φB124-14 terminase gene amino acid sequence. Homologous sequences, based on top bit scores, were identified in metagenomic datasets of human gut and marine origin [6], [8], [28], [60], [92], as well as through BlastP searches of the nr dataset. Sequences were aligned using ClustalW and the Neighbour-Joining method with the Jones-Taylor-Thornton matrix model for protein distance, used to construct phylogenetic trees using MEGA v5 [81]. Alignment-free analysis, based on the TRF patterns encoded in microbial and bacteriophage genomes, was used to investigate the broader relationship of φB124-14 with the wider phage community, and host bacterial species. Correlations between frequencies of all 256 possible tetranucleotide sequences in all phage genome sequences available in GenBank (611 phage genome sequences as of October 2011), a wide range of *Bacteroides* spp. genomes (48 genome sequences, obtained from GenBank, The Broad Institute – http://www.broadinstitute.org; and the Washington University Genome Institute – http://www.genome.wustl.edu), as well as all large metagenomic fragments (>10 kb) assembled from the human gut viral datasets generated by Reyes and colleagues [6], were calculated according to the method of Teeling and colleagues, using the standalone TETRA 1.0 program [65]. Draft *Bacteroides* chromosomal sequences were also included in this analysis and for each draft genome contigs were first concatenated before processing using TETRA (concatenation was confirmed not to obscure the inherent tetranucleotide genomes signature in draft genomes processed this way; Figure S2). All sequences entered into the TETRA standalone program were extended by their reverse complement and used by the program to calculate observed and expected TRFs [65]. The divergence between observed and expected frequencies for each tetranucleotide pattern were subsequently converted to Z-scores which were compared pairwise between all sequences to generate a Pearson similarity matrix of TRF patterns.

## Supporting Information

Figure S1 **Details of closest sequences to φB124-14 by tetra score.** For each sequence type represented (phage, virome, chromosome), the top six closest sequences to φB124-14 by tetranucleotide repeat frequency (TRF) score are indicated by numerals on the scatter plot, and colours correspond to sequence types (as detailed in chart legend). The table provides the names and TRF correlation values against the φB124-14 genome for each sequence indicated, arranged by sequence type. In the case of complete phage genome sequences, the closest sequence to φB124-14 is φB40-8 and vice versa.
(TIF)

Figure S2 **Comparison of tetranucleotide correlation scores for complete and draft concatenated genomes.** To verify that concatenation of draft genomes, and the unfinished nature of these datasets did not corrupt the tetranucleotide genome signatures of these genomes, complete and draft genomes for several Bacteroides species were compared. It is expected that such strains would exhibit a high level of correlation between tetranucleotide genome signatures. Scatter plots indicate that concatenated draft genomes retain their tetranucleotide signature, with perfect correlation observed in all comparisons, in contrast to negative control plots between the distantly related Bacteroides vulgatus and Bifidobacterium longum genomes. A. B. thetaiotaomicron VPI-5483 complete genome vs B. thetaiotaomicron 3330-1 draft concatenated genome. B. B. vulgatus ATCC 8482 complete genome vs B. vulgates 1_0 draft concatenated genome. C. B. fragilis YCH46 complete genome vs B. fragilis 3_1_12_1 draft concatenated genome. D. Negative control plot, B. fragilis YCH46 vs Bifidobacterium longum DJO10A. Corr  =  Correlation score.
(TIF)

Table S1 **Origin of species and strains used in φB124-14 host range assays^1^.** ^1^ highly related B. fragilis strains used for tree construction (Figure 1B) also included. NT – not tested.
(DOCX)

Table S2 **φB124-14 predicted ORFs and putative functional assignments.** ^1^ ORF numbers and functional assignments correspond to those represent on genetic maps of the ФB124-14 genome presented in **Figure 2** **.** ^2^ ORFs were assigned roles relating to broad functions based on results of BlastP and conserved domain searches of translated ORF amino acid sequences.
(DOCX)

Table S3 **Bacterial chromosomes, phage genomes and metagenomic fragments used in phage phylogenetic analyses and ecological profiling** (**Figures 7** **and** **8**).**1** – **Classification** **,** refers to classification of genomes used for ecological profiling in **Figure 8B** **.** Genomes from phage infecting host bacteria belonging from a particular genus were assigned one of three broad categories based on the relationship of bacterial host genus with the human gut microbiota. For the purposes of this analysis only bacteriophage with 4 or more representatives infecting a particular genus of bacteria were included (540 complete phage genomes, representing 31 bacterial genera). **G  =  Gut**, constitutes bacteriophage infecting genera commonly forming part of the normal human gut microbiota as well as all large fragments (>10 Kb) assembled using CAMERA workflows from human gut viral metagenomic libraries (Reyes *et al* 2010, *Nature* 466: 334–338 [6]). **GA  =  Gut Associated,** contains bacteriophage genomes infecting genera with member species associated with the gut but not considered to be members of the normal microbiota (such as primary invasive gut pathogens), and/or contain member species more commonly associated with environmental habitats. **NG  =  Non-Gut,** contains bacteriophage infecting genera with member species not considered to be members of the human gut microbiota or typically associated with this community. Primarily encompasses bacteriophage infecting genera of environmental origin. **2 – Source**, indicates the source of bacterial and bacteriophage genomes utilised in this study: **NCBI** – Complete bacteriophage genomes were obtained from the NCBI Viruses home page (TaxID: 10239) and all genomes present as of Oct 18th 2011 were downloaded using the Viral homepage ftp. Complete finished *Bacteroides* genomes were obtained from the NCBI Prokaryotes genome homepage and downloaded individually. • NCBI Viral Homepage: http://www.ncbi.nlm.nih.gov/genomes/GenomesHome.cgi?taxid=10239; • NCBI Viral FTP: ftp://ftp.ncbi.nih.gov/refseq/release/viral/; • NCBI Prokaryote Homepage: http://www.ncbi.nlm.nih.gov/genomes/lproks.cgi **. NCBI SRA** –Pyrosequencing reads generated from metagenomic libraries of virus-like particles by Reyes *et al*. (2010) [6], were obtained from the NCBI Short read archive, project SRA012183 (http://www.ncbi.nlm.nih.gov/sra). Reads were subsequently processed for quality and assembled using CAMERA workflows (https://portal.camera.calit2.net/gridsphere/gridsphere). **Broad Inst  =  Broad Institute.** Draft *Bacteroides spp*. genomes sequenced as part of the Human Microbiome Project (Nelson *et al* 2010 *Science* 328 (5981):994–999) at the Broad Institute were downloaded from the Bacteroides group Sequencing project page: • Broad Institute homepage (http://www.broadinstitute.org/); • Bacteroides Sequencing Group Project Page (http://www.broadinstitute.org/annotation/genome/bacteroides_group/MultiDownloads.html); • Human Microbiome Project Homepage (http://genome.wustl.edu/projects/human_microbiome_project/human_gut_microbiome). **WUGC**  =  **Washington University Genome Centre**. Draft Bacteroides genomes sequenced as part of the Human Gut Microbiome Project were also obtained from the Washington University Sequencing Centre, Human Microbiome Project website. • HGM Home page: http://genome.wustl.edu/projects/human_microbiome_project/human_microbiome_project_description. • Genomes: http://genome.wustl.edu/genomes/human_gut_microbiome_genomes.
(DOCX)
