# Supplementary figures and images for: Comparative (Meta)genomic Analysis and Ecological Profiling of Human Gut-Specific Bacteriophage φB124-14
Source: PLoS One. 2012 Apr 25;7(4):e35053. doi: 10.1371/journal.pone.0035053 (PMC3338817; doi:10.1371/journal.pone.0035053)

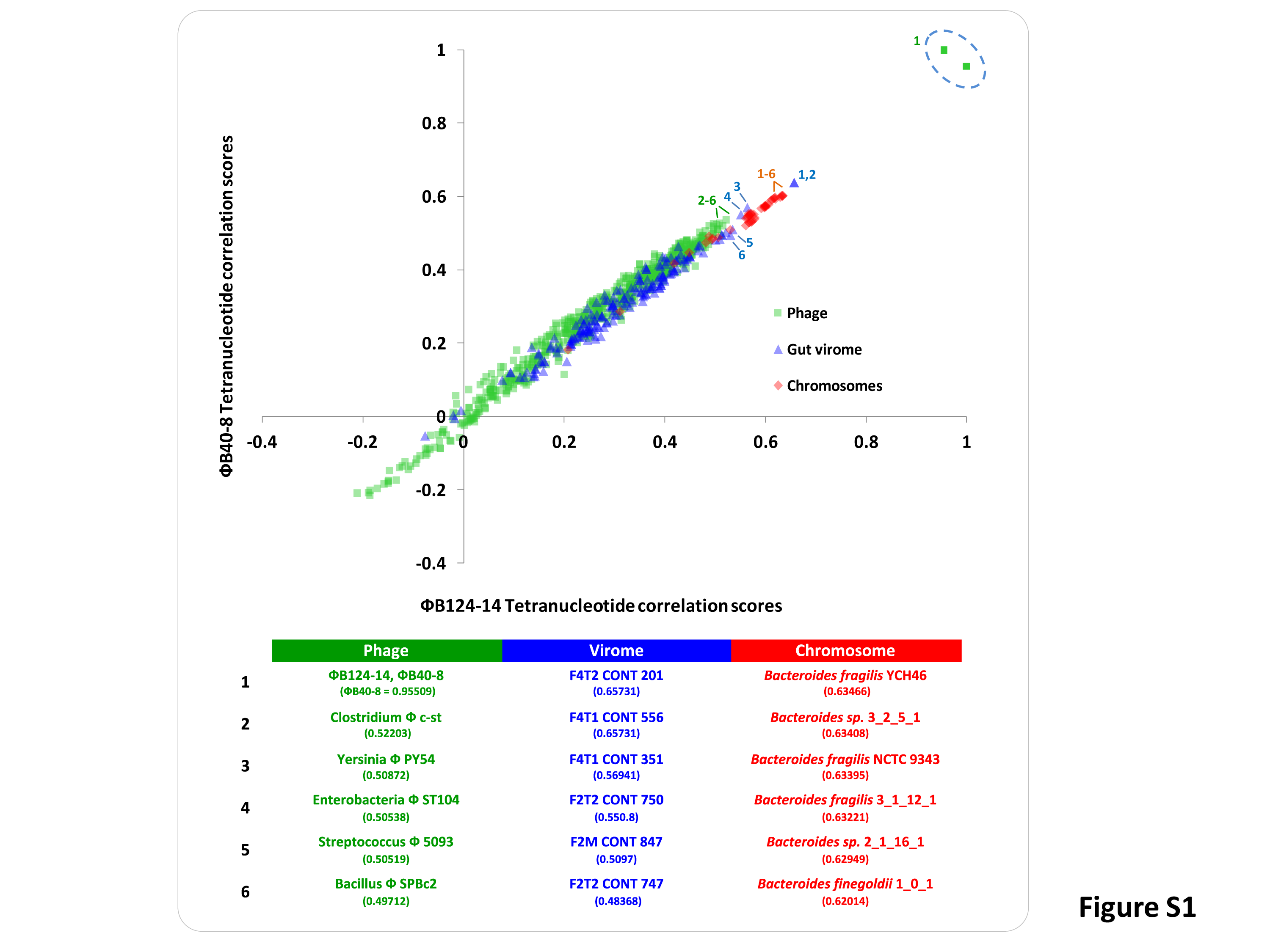

Supplement: Figure S1 — Details of closest sequences to φB124-14 by tetra score. For each sequence type represented (phage, virome, chromosome), the top six closest sequences to φB124-14 by tetranucleotide repeat frequency (TRF) score are indicated by numerals on the scatter plot, and colours correspond to sequence types (as detailed in chart legend). The table provides the names and TRF correlation values against the φB124-14 genome for each sequence indicated, arranged by sequence type. In the case of complete phage genome sequences, the closest sequence to φB124-14 is φB40-8 and vice versa. (TIF) [file pone.0035053.s001.tif]

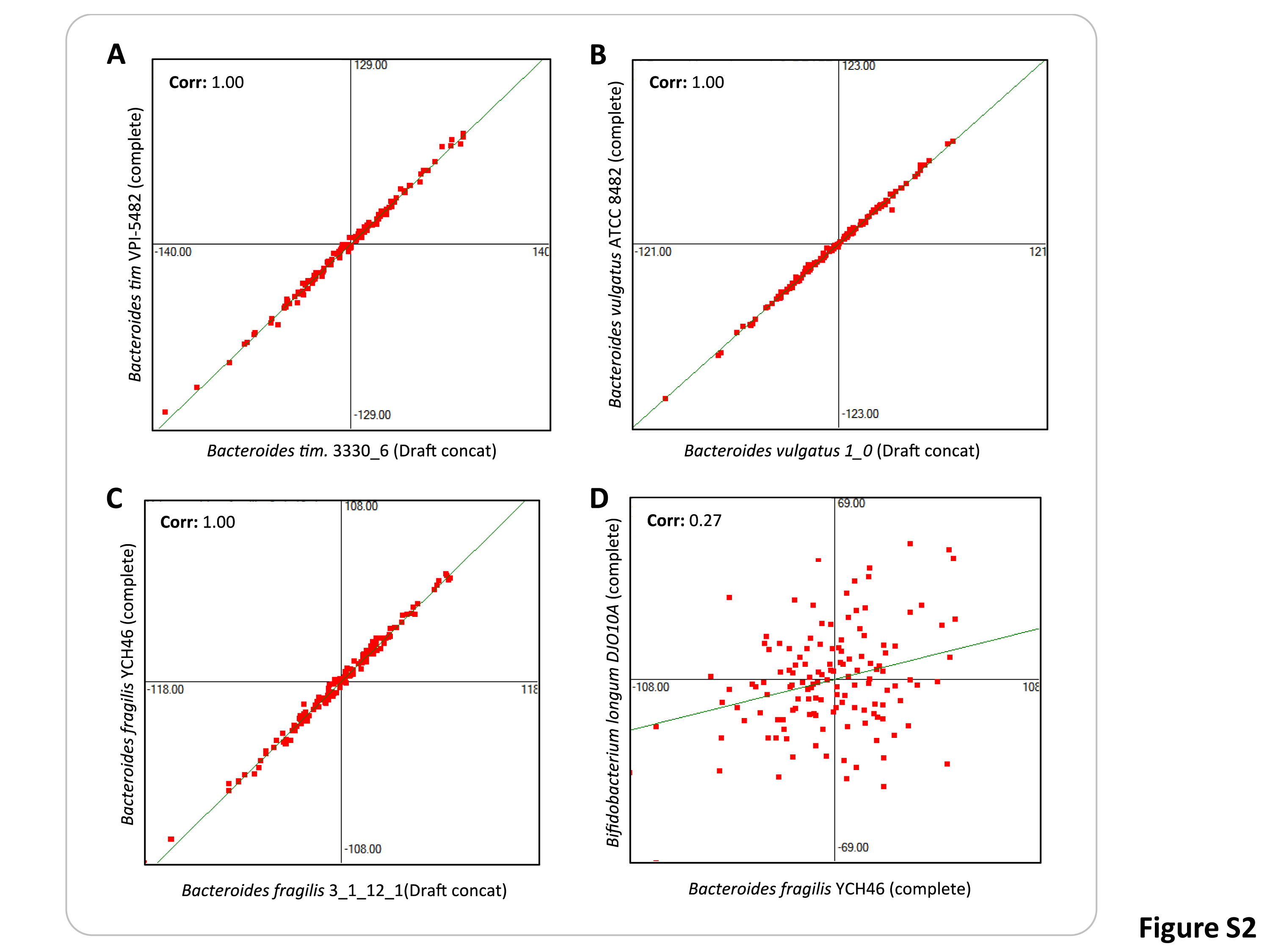

Supplement: Figure S2 — Comparison of tetranucleotide correlation scores for complete and draft concatenated genomes. To verify that concatenation of draft genomes, and the unfinished nature of these datasets did not corrupt the tetranucleotide genome signatures of these genomes, complete and draft genomes for several Bacteroides species were compared. It is expected that such strains would exhibit a high level of correlation between tetranucleotide genome signatures. Scatter plots indicate that concatenated draft genomes retain their tetranucleotide signature, with perfect correlation observed in all comparisons, in contrast to negative control plots between the distantly related Bacteroides vulgatus and Bifidobacterium longum genomes. A. B. thetaiotaomicron VPI-5483 complete genome vs B. thetaiotaomicron 3330-1 draft concatenated genome. B. B. vulgatus ATCC 8482 complete genome vs B. vulgates 1_0 draft concatenated genome. C. B. fragilis YCH46 complete genome vs B. fragilis 3_1_12_1 draft concatenated genome. D. Negative control plot, B. fragilis YCH46 vs Bifidobacterium longum DJO10A. Corr = Correlation score. (TIF) [file pone.0035053.s002.tif]
