# Supplementary material for: Comparative (Meta)genomic Analysis and Ecological Profiling of Human Gut-Specific Bacteriophage φB124-14
Source: PLoS One. 2012 Apr 25;7(4):e35053. doi: 10.1371/journal.pone.0035053 (PMC3338817; doi:10.1371/journal.pone.0035053)
Supplement: Table S1 — Origin of species and strains used in φB124-14 host range assays1. 1 highly related B. fragilis strains used for tree construction (Figure 1B) also included. NT – not tested. (DOCX) [file pone.0035053.s003.docx]

**Table S1: Origin of species and strains used in ɸB124-14 host range assays^1^**

^1^ highly related *B. fragilis* strains used for tree construction (Figure 1B) also included. NT – not tested.
