# Supplementary material for: Comparative (Meta)genomic Analysis and Ecological Profiling of Human Gut-Specific Bacteriophage φB124-14
Source: PLoS One. 2012 Apr 25;7(4):e35053. doi: 10.1371/journal.pone.0035053 (PMC3338817; doi:10.1371/journal.pone.0035053)
Supplement: Table S2 — φB124-14 predicted ORFs and putative functional assignments. 1 ORF numbers and functional assignments correspond to those represent on genetic maps of the ФB124-14 genome presented in Figure 2 . 2 ORFs were assigned roles relating to broad functions based on results of BlastP and conserved domain searches of translated ORF amino acid sequences. (DOCX) [file pone.0035053.s004.docx]

**Table S2: ϕB124-14 predicted ORFs and putative functional assignments**

| ORF^1^ | Start | End | Size (aa) | Predicted Function^2^ | Putative product^3^ | e value^3,4^ | % Identity^3,4^  (aa) |
| --- | --- | --- | --- | --- | --- | --- | --- |
| **1** | 182 | 48 | 44 | Unknown | Nsh | - | - |
| **2** | 509 | 192 | 106 | DNA replication and regulation | Putative exoribonuclease R (*Bacteroides* phage B40-8, B40-8021; YP_002221559.1) | 2.00E-44 | 67 (66/99) |
| **3** | 1456 | 512 | 315 | Unknown | Hypothetical protein (*Bacteroides* phage B40-8, B40-8020; YP_002221558.1) | 5.00E-171 | 94 (293/313) |
| **4** | 2139 | 1507 | 210 | DNA replication and regulation | Putative essential recombination protein (*Bacteroides* phage B40-8018; YP_002221556.1) | 7.00E-109 | 97 (190/196) |
| **5** | 2342 | 2145 | 65 | Unknown | Nsh | - | - |
| **6** | 2788 | 2393 | 131 | Unknown | Hypothetical protein (*Bacteroides* phage B40-8, B40-8017; YP_002221555.1) | 2.00E-62 | 92(120/131) |
| **7** | 3232 | 3059 | 57 | Unknown | Nsh | - | - |
| **8** | 4026 | 3229 | 265 | DNA replication and regulation | ThyA, Thymidylate synthase (*Bacteroides* phage B40-8, B40-8016; YP_002221554.1). Complete ThyA (pfam00303; TIGR3284) and pyrimidine synthase/hydroxymethylase conserved domains detected (cd00351). | 0 | 97 (255/265) |
| **9** | 4141 | 4028 | 37 | Unknown | Nsh | - | - |
| **10** | 4279 | 4163 | 38 | Unknown | Nsh | - | - |
| **11** | 5128 | 4292 | 278 | Unknown | Hypothetical protein (*Bacteroides* phage B40-8, B40-8014; YP_002221552.1). Partial conserved domains of unknown function detected (DUF1351 pfam07083). | 0 | 98 (272/278) |
| **12** | 5327 | 5130 | 65 | Unknown | Hypothetical protein (*Bacteroides* fragilis 3_1_12, Bfra3_11696; ZP_05281918.1). | 2.00E-04 | 38 (19/50) |
| **13** | 5790 | 5374 | 138 | Unknown | Hypothetical protein (*Bacteroides* phage B40-8, B40-8013; YP_002221551.1). | 2.00E-70 | 95 (130/138) |
| **14** | 6089 | 5808 | 93 | Unknown | Nsh | - |  |
| **15** | 6353 | 6108 | 81 | Unknown | Hypothetical protein (*Bacteroides* phageB40-8, B40-8012; YP_002221550.1). | 2.00E-36 | 92 (74/81) |
| **16** | 7370 | 6765 | 201 | Unknown | Hypothetical protein (*Bacteroides* phage B40-8, B40-8011; YP_002221549.1). | 1.00E-85 | 79 (57/201) |
| **17** | 7762 | 7367 | 131 | Lysis | Putative peptidase (*Bacteroides* phage B40-8, B40-8010; YP_002221548.1). Complete conserved domains from M15_3 type peptidase detected (pfam08291) and partial domains from M15_2 and uncharacterised bacterial proteins detected (pfam05951 and COG3108 respectively). | 6.00E-52 | 74 (96/131) |
| **18** | 8296 | 7805 | 163 | Unknown | Hypothetical protein (*Bacteroides* phage B40-8, B40-8009; YP_002221547.1). | 9.00E-44 | 70 (79/113) |
| **19** | 8664 | 8296 | 122 | Unknown | Hypothetical protein (*Bacteroides* phage B40-8, B40-8008; YP_002221546.1). | 3.00E-28 | 53 (61/117) |
| **20** | 14766 | 8746 | 2006 | Structure | Putative phage tail fibre protein (*Bacteroides* phage B40-8, B40-8007; (YP_002221545.1). | 0 | 75 (1441/1929) |
| **21** | 17000 | 14826 | 724 | Unknown | Hypothetical protein (*Bacteroides* phage B40-8, B40-8005; YP_002221543.1). | 0 | 91 (555/611) |
| **22** | 20125 | 17000 | 1041 | DNA replication and regulation | Putative DNA segregation protein similar to B40-8003 hypothetical protein from *Bacteroides* phage B40-8 (YP_002221541.1). Partial conserved domains from SMC_prok_B (TIGR02168) bacterial chromosome segregation proteins detected | 0 | 81 (851/1054) |
| **23** | 20889 | 20137 | 250 | Unknown | Hypothetical protein (*Bacteroides* phage B40-8, B40-8002; YP_002221543.1). | 2.00E-144 | 99 (247/250) |
| **24** | 21163 | 20876 | 95 | Unknown | Nsh | - | - |
| **25** | 21468 | 21163 | 101 | Unknown | Nsh | - | - |
| **26** | 21990 | 21610 | 126 | Unknown | Nsh | - | - |
| **27** | 22339 | 21983 | 118 | Unknown | Hypothetical protein (*Bacteroides* phage B40-8, B40-8001; YP_002221543.1). | 1.00E-24 | 55 (60/110) |
| **28** | 22611 | 22402 | 69 | Unknown | Nsh | - | - |
| **29** | 23079 | 22615 | 154 | Unknown | Nsh | - | - |
| **30** | 23348 | 23076 | 90 | Unknown | Nsh | - | - |
| **31** | 23791 | 23546 | 81 | Unknown | Nsh | - | - |
| **32** | 24044 | 23784 | 86 | Unknown | Nsh | - | - |
| **33** | 24058 | 24192 | 44 | Unknown | Nsh | - | - |
| **34** | 24469 | 24329 | 46 | Unknown | Hypothetical protein (*Bacteroides* *stercoris* ATCC 43183, BACSTE_02177; ZP_02435924.1). | 9.00E-11 | 63 (29/46) |
| **35** | 25168 | 24599 | 189 | Unknown | Hypothetical protein (*Bacteroides* phage B40-8, B40-8046; YP_002221543.1). | 1.00E-137 | 99 (186/189) |
| **36** | 25610 | 25158 | 150 | Unknown | Hypothetical protein (*Bacteroides* phage B40-8, B40-8045; YP_002221543.1). | 1.00E-80 | 100 (150/150) |
| **37** | 26920 | 25607 | 437 | Unknown | Hypothetical protein (*Bacteroides* phage B40-8, B40-8044; YP_002221543.1). | 0 | 97 (423/437) |
| **38** | 28233 | 26989 | 414 | Structure | Putative capsid protein; similar to major protein 1 (MP1, B40-8043) from *Bacteroides* phage B40-8 (YP_002221581.1). | 0 | 96 (396/414) |
| **39** | 29273 | 28356 | 305 | Structure | Capsid associated protein. Similar to hypothetical protein from *Bacteroides* phage B40-8, B40-8042; YP_002221543.1). | 0 | 99 (303/305) |
| **40** | 29671 | 29273 | 132 | Unknown | Hypothetical protein (*Bacteroides* phage B40-8, B40-8041; YP_002221543.1). | 1.00E-59 | 97 (128/132) |
| **41** | 31373 | 29634 | 577 | Structure | Putative capsid protein; similar to major protein 3 (MP3, B40-8040) from *Bacteroides* phage B40-8 (YP_002221578.1). | 0 | 99 (574/577) |
| **42** | 32153 | 31515 | 210 | Structure | Putative capsid protein; similar to major protein 2 (MP2, B40-8039) from *Bacteroides* phage B40-8 (YP_002221577.1). | 9.00E-148 | 96 (201/210) |
| **43** | 34741 | 32240 | 833 | Packaging | Putative phage terminase large subunit. Similar to B40-8038 from Bacteroides phage B40-8 (YP_002221576.1). Complete conserved domains from uncharacterised putative phage terminase proteins (TIGR01630) detected. Partial conserved domains from uncharacterised bacterial proteins (COG5410) and homing endonucleases (pfam0502, pfam0503). | 0 | 97 (382/397) |
| **44** | 35307 | 34738 | 189 | Unknown | Hypothetical protein (*Bacteroides* phage B40-8, B40-8037; YP_002221543.1). | 5.00E-107 | 99 (187/189) |
| **45** | 36150 | 35485 | 221 | Unknown | Hypothetical protein (*Bacteroides* phage B40-8, B40-8036; YP_002221543.1). Complete conserved domains from bacteriophage proteins of unknown function detected DUF3310 (pfam11753). | 2.00E-109 | 91 (199/221) |
| **46** | 36748 | 36161 | 201 | Unknown | Hypothetical protein (*Bacteroides* phage B40-8, B40-8035; YP_002221543.1). | 8.00E-43 | 52 (104/201) |
| **47** | 37074 | 36772 | 100 | Unknown | Hypothetical protein (*Bacteroides* phage B40-8, B40-8034; YP_002221543.1). | 3.00E-34 | 77 (77/100) |
| **48** | 37276 | 37061 | 71 | Unknown | Nsh | - | - |
| **49** | 37643 | 37269 | 124 | Unknown | Hypothetical protein (*Bacteroides* phage B40-8, B40-8033; YP_002221543.1). | 7.00E-61 | 91 (112/124) |
| **50** | 37913 | 37680 | 77 | Unknown | Nsh | - | - |
| **51** | 38520 | 38065 | 151 | DNA replication and regulation | Putative single-strand DNA binding protein (*Bacteroides* phage B40-8, B40-8032; YP_002221570.1). Complete SSB_OBF conserved domains detected (cd04496) and partial single stranded binding protein domains detected (pfam00436;TIGR00621;COG0629). | 1.00E-96 | 92 (138/151) |
| **52** | 38783 | 38520 | 87 | Unknown | Nsh | - | - |
| **53** | 39211 | 38780 | 143 | DNA replication and regulation | Putative HNH endonuclease. Similar to B40-8031 from *Bacteroides* phage B40-8 (YP_002221569.1). Complete HNH endonuclease conserved domains detected (pfam01844), and partial PBP1_RegR_EndR_KdgR_like ligand-binding domain of transcriptional repressors detected (cd06238). | 4.00E-80 | 99 (142/143) |
| **54** | 39848 | 39198 | 216 | Unknown | Hypothetical protein (*Bacteroides* phage B40-8, B40-8030; YP_002221543.1). | 6.00E-113 | 94 (202/216) |
| **55** | 40311 | 39997 | 104 | Unknown | Hypothetical protein (*Bacteroides* phage B40-8, B40-8029; YP_002221543.1). | 3.00E-53 | 99 (102/104) |
| **56** | 40745 | 40308 | 145 | Unknown | Hypothetical protein (*Bacteroides* phage B40-8, B40-8028; YP_002221543.1). | 4.00E-77 | 96 (138/145) |
| **57** | 40881 | 40705 | 58 | Unknown | Hypothetical protein (*Bacteroides* phage B40-8, B40-8027; YP_002221543.1). | 3.00E-21 | 88 (51/58) |
| **58** | 41365 | 41186 | 59 | Unknown | Nsh | - | - |
| **59** | 41939 | 41325 | 204 | Unknown | Hypothetical protein (*Bacteroides* phage B40-8, B40-8025; YP_002221543.1). | 1.00E-133 | 55 (104/188) |
| **60** | 42151 | 41930 | 73 | Unknown | Nsh | - | - |
| **61** | 43107 | 42163 | 314 | Unknown | Hypothetical protein (*Bacteroides* phage B40-8, B40-8024; YP_002221543.1). | 3.00E-98 | 62 (179/292) |
| **62** | 44108 | 43158 | 316 | Unknown | Hypothetical protein (*Bacteroides* phage B40-8, B40-8023; YP_002221543.1). | 4.00E-58 | 70 (120/172) |
| **63** | 44325 | 44158 | 55 | DNA replication and regulation | Putative phage antirepressor. Similar to predicted protein *Bacteroides* sp. 2_2_4 (ZP_04551591.1). | 1.00E-05 | 86 (23/27) |
| **64** | 44617 | 44372 | 81 | DNA replication and regulation | Putative mismatch repair protein. Similar to hypothetical protein PARMER_02659 from *Parabacteroides* *merdae* ATCC 43184 (ZP_02032642.1). Partial MutS mismatch repair conserved domains detected (pfam01624, PRK05399, TIGR01070,COG0249). | 4.00E-19 | 49 (39/81) |
| **65** | 45313 | 44660 | 217 | DNA replication and regulation | Putative resolvase/recombinase. Similar to multiple promoter invertase from *Bacteroides* sp. 3_2_5 (ZP_04840901.1), and resolvase from *Bacteroides ovatus* SD CC 2a (ZP_06722395.1). Complete domains from serine recombinase type proteins (PRK13413, pfam00239,cd03768,smart00857), and Helix_turn_helix Hin_like domains detected (cd00569). | 9E-113 | 93 (201/217) |
| **66** | 45709 | 45323 | 131 | DNA replication and regulation | Putative nuclease. Similar to hypothetical protein Bfra3_07147 from *Bacteroides* *fragilis* 3_1_12 (ZP_05281024.1). Complete IERN1 intron encoded nuclease repeat motif and NUMOD1 domains detected (smart00479). | 5.00E-30 | 50 (65/131) |
| **67** | 46529 | 45774 | 252 | DNA replication and regulation | Putative phage anitrepressor. Similar to antirepressor from *lactobacillus* phage Lrm1 (YP_002117696.1), *L. rhamnosus* Lc 705m (YP_003173532.1) and Bacteriodes phage B40-8 (B40-8022, YP_002221560.1). Complete phage_pRha regulatory domain (pfam09669) and ANT KilAC domain detected (pfam03374). | 7.00E-59 | 54 (134/252) |
| **68** | 46763 | 46554 | 69 | Unknown | Nsh | - | - |

**^1^** ORF numbers and functional assignments correspond to those represent on genetic maps of the ФB124-14 genome presented in **Figure 2**

**^2^** ORFs were assigned roles relating to broad functions based on results of BlastP and conserved domain searches of translated ORF amino acid sequences.

**^3^** Putative products of predicted ORFs were designated based on BlastP and conserved domain searches. % identity and e-values are shown for top hits in BlastP searches. e-values for hits to conserved domains were 4e^-03^ or lower. nsh indicates no significant hits detected for a particular amino acid sequence.

**^4^** Percent identity for top hits in BlastP searches. Figures in parentheses provide length of each alignment.
