# Supplementary material for: Comparative (Meta)genomic Analysis and Ecological Profiling of Human Gut-Specific Bacteriophage φB124-14
Source: PLoS One. 2012 Apr 25;7(4):e35053. doi: 10.1371/journal.pone.0035053 (PMC3338817; doi:10.1371/journal.pone.0035053)
Supplement: Table S3 — Bacterial chromosomes, phage genomes and metagenomic fragments used in phage phylogenetic analyses and ecological profiling ( Figures 7 and 8 ).1 – Classification , refers to classification of genomes used for ecological profiling in Figure 8B . Genomes from phage infecting host bacteria belonging from a particular genus were assigned one of three broad categories based on the relationship of bacterial host genus with the human gut microbiota. For the purposes of this analysis only bacteriophage with 4 or more representatives infecting a particular genus of bacteria were included (540 complete phage genomes, representing 31 bacterial genera). G = Gut, constitutes bacteriophage infecting genera commonly forming part of the normal human gut microbiota as well as all large fragments (>10 Kb) assembled using CAMERA workflows from human gut viral metagenomic libraries (Reyes et al 2010, Nature 466: 334–338 [6]). GA = Gut Associated, contains bacteriophage genomes infecting genera with member species associated with the gut but not considered to be members of the normal microbiota (such as primary invasive gut pathogens), and/or contain member species more commonly associated with environmental habitats. NG = Non-Gut, contains bacteriophage infecting genera with member species not considered to be members of the human gut microbiota or typically associated with this community. Primarily encompasses bacteriophage infecting genera of environmental origin. 2 – Source, indicates the source of bacterial and bacteriophage genomes utilised in this study: NCBI – Complete bacteriophage genomes were obtained from the NCBI Viruses home page (TaxID: 10239) and all genomes present as of Oct 18th 2011 were downloaded using the Viral homepage ftp. Complete finished Bacteroides genomes were obtained from the NCBI Prokaryotes genome homepage and downloaded individually. • NCBI Viral Homepage: http://www.ncbi.nlm.nih.gov/genomes/GenomesHome.cgi?taxid=10239; • NCBI Viral FTP: ftp://ftp.ncbi [file pone.0035053.s005.docx]

**Table S3: Bacterial chromosomes, phage genomes and metagenomic fragments used in phage phylogenetic analyses and ecological profiling (Figures 7 and 8).**

**1** – **Classification,** refers to classification of genomes used for ecological profiling in **Figure 8B**. Genomes from phage infecting host bacteria belonging from a particular genus were assigned one of three broad categories based on the relationship of bacterial host genus with the human gut microbiota. For the purposes of this analysis only bacteriophage with 4 or more representatives infecting a particular genus of bacteria were included (540 complete phage genomes, representing 31 bacterial genera).

**G = Gut**, constitutes bacteriophage infecting genera commonly forming part of the normal human gut microbiota as well as all large fragments (>10 Kb) assembled using CAMERA workflows from human gut viral metagenomic libraries (Reyes *et al* 2010, *Nature* 466: 334-338 [6]).

**GA = Gut Associated,** contains bacteriophage genomes infecting genera with member species associated with the gut but not considered to be members of the normal microbiota (such as primary invasive gut pathogens), and/or contain member species more commonly associated with environmental habitats.

**NG = Non-Gut,** contains bacteriophage infecting genera with member species not considered to be members of the human gut microbiota or typically associated with this community. Primarily encompasses bacteriophage infecting genera of environmental origin.

**2 – Source**, indicates the source of bacterial and bacteriophage genomes utilised in this study:

# NCBI – Complete bacteriophage genomes were obtained from the NCBI Viruses home page (TaxID: 10239) and all genomes present as of Oct 18^th^ 2011 were downloaded using the Viral homepage ftp. Complete finished *Bacteroides* genomes were obtained from the NCBI Prokaryotes genome homepage and downloaded individually.

# NCBI Viral Homepage: <http://www.ncbi.nlm.nih.gov/genomes/GenomesHome.cgi?taxid=10239>

# NCBI Viral FTP: <ftp://ftp.ncbi.nih.gov/refseq/release/viral/>

# NCBI Prokaryote Homepage: <http://www.ncbi.nlm.nih.gov/genomes/lproks.cgi>

**NCBI SRA** –Pyrosequencing reads generated from metagenomic libraries of virus-like particles by Reyes *et al.* (2010) [6], were obtained from the NCBI Short read archive, project SRA012183 (<http://www.ncbi.nlm.nih.gov/sra>). Reads were subsequently processed for quality and assembled using CAMERA workflows (<https://portal.camera.calit2.net/gridsphere/gridsphere>).

**Broad Inst = Broad Institute**. Draft *Bacteroides* *spp*. genomes sequenced as part of the Human Microbiome Project (Nelson *et* *al* 2010 *Science* 328 (5981):994-999) at the Broad Institute were downloaded from the Bacteroides group Sequencing project page:

- Broad Institute homepage (<http://www.broadinstitute.org/>)
- Bacteroides Sequencing Group Project Page (<http://www.broadinstitute.org/annotation/genome/bacteroides_group/MultiDownloads.html>)
- Human Microbiome Project Homepage (<http://genome.wustl.edu/projects/human_microbiome_project/human_gut_microbiome>)

**WUGC** = **Washington University Genome Centre**. Draft Bacteroides genomes sequenced as part of the Human Gut Microbiome Project were also obtained from the Washington University Sequencing Centre, Human Microbiome Project website.

- HGM Home page: <http://genome.wustl.edu/projects/human_microbiome_project/human_microbiome_project_description>
- Genomes: <http://genome.wustl.edu/genomes/human_gut_microbiome_genomes>

| **Sequence/Genome** | **Type** | **Status** | **Classification^1^** | **Source^2^** |
| --- | --- | --- | --- | --- |
| Bacteroides fragilis B124-14 | Phage | Complete | G | **This study (HE608841)** |
| Bacteroides fragilis B40-8 | Phage | Complete | G | NCBI |
| Acholeplasma_phage_L2_complete_genome. | Phage | Complete | - | NCBI |
| Acholeplasma_phage_MV-L1_complete_genome. | Phage | Complete | - | NCBI |
| Acinetobacter_phage_133_complete_genome. | Phage | Complete | NG | NCBI |
| Acinetobacter_phage_Ac42_complete_genome. | Phage | Complete | NG | NCBI |
| Acinetobacter_phage_Acj61_complete_genome. | Phage | Complete | NG | NCBI |
| Acinetobacter_phage_Acj9_complete_genome. | Phage | Complete | NG | NCBI |
| Acinetobacter_phage_AP205_complete_genome. | Phage | Complete | NG | NCBI |
| Actinomyces_phage_Av-1_complete_genome. | Phage | Complete | - | NCBI |
| Actinoplanes_phage_phiAsp2_complete_genome. | Phage | Complete | - | NCBI |
| Acyrthosiphon_pisum_bacteriophage_APSE-1_complete_genome. | Phage | Complete | - | NCBI |
| Aeromonas_phage_25_complete_genome. | Phage | Complete | NG | NCBI |
| Aeromonas_phage_31_complete_genome. | Phage | Complete | NG | NCBI |
| Aeromonas_phage_44RR2.8t_complete_genome. | Phage | Complete | NG | NCBI |
| Aeromonas_phage_65_complete_genome. | Phage | Complete | NG | NCBI |
| Aeromonas_phage_Aeh1_complete_genome. | Phage | Complete | NG | NCBI |
| Aeromonas_phage_phiAS4_complete_genome. | Phage | Complete | NG | NCBI |
| Aeromonas_phage_phiAS5_complete_genome. | Phage | Complete | NG | NCBI |
| Aeromonas_phage_phiO18P_complete_genome. | Phage | Complete | NG | NCBI |
| Aggregatibacter_phage_S1249_complete_genome. | Phage | Complete | - | NCBI |
| Azospirillum_phage_Cd_complete_genome. | Phage | Complete | - | NCBI |
| Bacillus_phage_0305phi8-36_complete_genome. | Phage | Complete | GA | NCBI |
| Bacillus_phage_AP50_complete_genome. | Phage | Complete | GA | NCBI |
| Bacillus_phage_B103_complete_genome. | Phage | Complete | GA | NCBI |
| Bacillus_phage_Bam35c_complete_genome. | Phage | Complete | GA | NCBI |
| Bacillus_phage_BCJA1c_complete_genome. | Phage | Complete | GA | NCBI |
| Bacillus_phage_Cherry_complete_genome. | Phage | Complete | GA | NCBI |
| Bacillus_phage_Fah_complete_genome. | Phage | Complete | GA | NCBI |
| Bacillus_phage_GA-1_complete_genome. | Phage | Complete | GA | NCBI |
| Bacillus_phage_Gamma_complete_genome. | Phage | Complete | GA | NCBI |
| Bacillus_phage_GIL16c_complete_genome. | Phage | Complete | GA | NCBI |
| Bacillus_phage_IEBH_complete_genome. | Phage | Complete | GA | NCBI |
| Bacillus_phage_phi105_complete_genome. | Phage | Complete | GA | NCBI |
| Bacillus_phage_phi29_complete_genome. | Phage | Complete | GA | NCBI |
| Bacillus_phage_SPBc2_complete_genome. | Phage | Complete | GA | NCBI |
| Bacillus_phage_SPO1_complete_genome. | Phage | Complete | GA | NCBI |
| Bacillus_phage_SPP1_complete_genome. | Phage | Complete | GA | NCBI |
| Bacillus_phage_TP21-L_complete_genome. | Phage | Complete | GA | NCBI |
| Bacillus_phage_WBeta_complete_genome. | Phage | Complete | GA | NCBI |
| Bacillus_prophage_phBC6A51_complete_genome. | Phage | Complete | GA | NCBI |
| Bacillus_prophage_phBC6A52_complete_genome. | Phage | Complete | GA | NCBI |
| Bacillus_virus_1_complete_genome. | Phage | Complete | GA | NCBI |
| Bacteriophage_Aaphi23_complete_genome. | Phage | Complete | - | NCBI |
| Bacteriophage_APSE-2_complete_genome. | Phage | Complete | - | NCBI |
| Bacteriophage_PSA_complete_genome. | Phage | Complete | - | NCBI |
| Bacteroides_phage_B40-8_complete_genome. | Phage | Complete | - | NCBI |
| Bdellovibrio_phage_phi1402_complete_genome. | Phage | Complete | - | NCBI |
| Bdellovibrio_phage_phiMH2K_complete_genome. | Phage | Complete | - | NCBI |
| Bordetella_phage_BIP-1_complete_genome. | Phage | Complete | - | NCBI |
| Bordetella_phage_BMP-1_complete_genome. | Phage | Complete | - | NCBI |
| Bordetella_phage_BPP-1_complete_genome. | Phage | Complete | - | NCBI |
| Brochothrix_phage_A9_complete_genome. | Phage | Complete | - | NCBI |
| Brochothrix_phage_BL3_complete_genome. | Phage | Complete | - | NCBI |
| Brochothrix_phage_NF5_complete_genome. | Phage | Complete | - | NCBI |
| Burkholderia_ambifaria_phage_BcepF1_complete_genome. | Phage | Complete | NG | NCBI |
| Burkholderia_phage_Bcep1_complete_genome. | Phage | Complete | NG | NCBI |
| Burkholderia_phage_Bcep176_complete_genome. | Phage | Complete | NG | NCBI |
| Burkholderia_phage_Bcep22_complete_genome. | Phage | Complete | NG | NCBI |
| Burkholderia_phage_Bcep43_complete_genome. | Phage | Complete | NG | NCBI |
| Burkholderia_phage_Bcep781_complete_genome. | Phage | Complete | NG | NCBI |
| Burkholderia_phage_BcepB1A_complete_genome. | Phage | Complete | NG | NCBI |
| Burkholderia_phage_BcepC6B_complete_genome. | Phage | Complete | NG | NCBI |
| Burkholderia_phage_BcepGomr_complete_genome. | Phage | Complete | NG | NCBI |
| Burkholderia_phage_BcepIL02_complete_genome. | Phage | Complete | NG | NCBI |
| Burkholderia_phage_BcepMu_complete_genome. | Phage | Complete | NG | NCBI |
| Burkholderia_phage_BcepNazgul_complete_genome. | Phage | Complete | NG | NCBI |
| Burkholderia_phage_BcepNY3_complete_genome. | Phage | Complete | NG | NCBI |
| Burkholderia_phage_KL3_complete_genome. | Phage | Complete | NG | NCBI |
| Burkholderia_phage_KS10_complete_genome. | Phage | Complete | NG | NCBI |
| Burkholderia_phage_KS14_complete_genome. | Phage | Complete | NG | NCBI |
| Burkholderia_phage_KS5_complete_genome. | Phage | Complete | NG | NCBI |
| Burkholderia_phage_KS9_complete_genome. | Phage | Complete | NG | NCBI |
| Burkholderia_phage_phi1026b_complete_genome. | Phage | Complete | NG | NCBI |
| Burkholderia_phage_phi644-2_chromosome_complete_genome. | Phage | Complete | NG | NCBI |
| Burkholderia_phage_phiE12-2_chromosome_complete_genome. | Phage | Complete | NG | NCBI |
| Burkholderia_phage_phiE125_complete_genome. | Phage | Complete | NG | NCBI |
| Burkholderia_phage_phiE202_chromosome_complete_genome. | Phage | Complete | NG | NCBI |
| Burkholderia_phage_phiE255_chromosome_complete_genome. | Phage | Complete | NG | NCBI |
| Burkholderia_prophage_phi52237_complete_genome. | Phage | Complete | NG | NCBI |
| Campylobacter_phage_NCTC12673_complete_genome. | Phage | Complete | - | NCBI |
| Chlamydia_phage_3_complete_genome. | Phage | Complete | NG | NCBI |
| Chlamydia_phage_4_complete_genome. | Phage | Complete | NG | NCBI |
| Chlamydia_phage_Chp1_complete_genome. | Phage | Complete | NG | NCBI |
| Chlamydia_phage_Chp2_complete_genome. | Phage | Complete | NG | NCBI |
| Chlamydia_phage_CPAR39_complete_genome. | Phage | Complete | NG | NCBI |
| Chlamydia_phage_PhiCPG1_complete_genome. | Phage | Complete | NG | NCBI |
| Clavibacter_phage_CMP1_complete_genome. | Phage | Complete | - | NCBI |
| Clostridium_phage_39-O_complete_genome. | Phage | Complete | G | NCBI |
| Clostridium_phage_c-st_complete_genome. | Phage | Complete | G | NCBI |
| Clostridium_phage_phi_CD119_complete_genome. | Phage | Complete | G | NCBI |
| Clostridium_phage_phi3626_complete_genome. | Phage | Complete | G | NCBI |
| Clostridium_phage_phiC2_complete_genome. | Phage | Complete | G | NCBI |
| Clostridium_phage_phiCD27_complete_genome. | Phage | Complete | G | NCBI |
| Clostridium_phage_phiCD38-2_complete_genome. | Phage | Complete | G | NCBI |
| Clostridium_phage_phiCD6356_complete_genome. | Phage | Complete | G | NCBI |
| Clostridium_phage_phiCTP1_complete_genome. | Phage | Complete | G | NCBI |
| Clostridium_phage_phiSM101_chromosome_complete_genome. | Phage | Complete | G | NCBI |
| Corynebacterium_phage_BFK20_complete_genome. | Phage | Complete | - | NCBI |
| Corynebacterium_phage_P1201_complete_genome. | Phage | Complete | - | NCBI |
| Cyanophage_PSS2_complete_genome. | Phage | Complete | - | NCBI |
| Cyanophage_Syn5_complete_genome. | Phage | Complete | - | NCBI |
| Deftia_phage_phiW-14_complete_genome. | Phage | Complete | - | NCBI |
| Enterobacteria_phage_13a_complete_genome. | Phage | Complete | G | NCBI |
| Enterobacteria_phage_285P_complete_genome. | Phage | Complete | G | NCBI |
| Enterobacteria_phage_933W_complete_genome. | Phage | Complete | G | NCBI |
| Enterobacteria_phage_alpha3_complete_genome. | Phage | Complete | G | NCBI |
| Enterobacteria_phage_BA14_complete_genome. | Phage | Complete | G | NCBI |
| Enterobacteria_phage_BP-4795_complete_genome. | Phage | Complete | G | NCBI |
| Enterobacteria_phage_CC31_complete_genome. | Phage | Complete | G | NCBI |
| Enterobacteria_phage_EcoDS1_complete_genome. | Phage | Complete | G | NCBI |
| Enterobacteria_phage_EPS7_complete_genome. | Phage | Complete | G | NCBI |
| Enterobacteria_phage_epsilon15_complete_genome. | Phage | Complete | G | NCBI |
| Enterobacteria_phage_ES18_complete_genome. | Phage | Complete | G | NCBI |
| Enterobacteria_phage_Felix_01_complete_genome. | Phage | Complete | G | NCBI |
| Enterobacteria_phage_Fels-2_complete_genome. | Phage | Complete | G | NCBI |
| Enterobacteria_phage_FI_complete_genome. | Phage | Complete | G | NCBI |
| Enterobacteria_phage_G4_complete_genome. | Phage | Complete | G | NCBI |
| Enterobacteria_phage_GA_complete_genome. | Phage | Complete | G | NCBI |
| Enterobacteria_phage_HK022_complete_genome. | Phage | Complete | G | NCBI |
| Enterobacteria_phage_HK620_complete_genome. | Phage | Complete | G | NCBI |
| Enterobacteria_phage_HK97_complete_genome. | Phage | Complete | G | NCBI |
| Enterobacteria_phage_I2-2_complete_genome. | Phage | Complete | G | NCBI |
| Enterobacteria_phage_ID18_complete_genome. | Phage | Complete | G | NCBI |
| Enterobacteria_phage_ID2_Moscow_ID_2001_complete_genome. | Phage | Complete | G | NCBI |
| Enterobacteria_phage_If1_complete_genome. | Phage | Complete | G | NCBI |
| Enterobacteria_phage_Ike_complete_genome. | Phage | Complete | G | NCBI |
| Enterobacteria_phage_IME08_complete_genome. | Phage | Complete | G | NCBI |
| Enterobacteria_phage_JK06_complete_genome. | Phage | Complete | G | NCBI |
| Enterobacteria_phage_JS10_complete_genome. | Phage | Complete | G | NCBI |
| Enterobacteria_phage_JS98_complete_genome. | Phage | Complete | G | NCBI |
| Enterobacteria_phage_JSE_complete_genome. | Phage | Complete | G | NCBI |
| Enterobacteria_phage_K1-5_complete_genome. | Phage | Complete | G | NCBI |
| Enterobacteria_phage_K1E_complete_genome. | Phage | Complete | G | NCBI |
| Enterobacteria_phage_K1F_complete_genome. | Phage | Complete | G | NCBI |
| Enterobacteria_phage_K30_complete_genome. | Phage | Complete | G | NCBI |
| Enterobacteria_phage_lambda_complete_genome. | Phage | Complete | G | NCBI |
| Enterobacteria_phage_LKA1_complete_genome. | Phage | Complete | G | NCBI |
| Enterobacteria_phage_M13_complete_genome. | Phage | Complete | G | NCBI |
| Enterobacteria_phage_Min27_complete_genome. | Phage | Complete | G | NCBI |
| Enterobacteria_phage_Mu_complete_genome. | Phage | Complete | G | NCBI |
| Enterobacteria_phage_N15_complete_genome. | Phage | Complete | G | NCBI |
| Enterobacteria_phage_N4_complete_genome. | Phage | Complete | G | NCBI |
| Enterobacteria_phage_P1_complete_genome. | Phage | Complete | G | NCBI |
| Enterobacteria_phage_P2_complete_genome. | Phage | Complete | G | NCBI |
| Enterobacteria_phage_P22_virus_complete_genome. | Phage | Complete | G | NCBI |
| Enterobacteria_phage_P4_complete_genome. | Phage | Complete | G | NCBI |
| Enterobacteria_phage_Phi1_complete_genome. | Phage | Complete | G | NCBI |
| Enterobacteria_phage_phiEco32_complete_genome. | Phage | Complete | G | NCBI |
| Enterobacteria_phage_phiEcoM-GJ1_complete_genome. | Phage | Complete | G | NCBI |
| Enterobacteria_phage_phiP27_complete_genome. | Phage | Complete | G | NCBI |
| Enterobacteria_phage_phiX174_complete_genome. | Phage | Complete | G | NCBI |
| Enterobacteria_phage_PRD1_complete_genome. | Phage | Complete | G | NCBI |
| Enterobacteria_phage_PsP3_complete_genome. | Phage | Complete | G | NCBI |
| Enterobacteria_phage_Qbeta_complete_genome. | Phage | Complete | G | NCBI |
| Enterobacteria_phage_RB14_complete_genome. | Phage | Complete | G | NCBI |
| Enterobacteria_phage_RB16_complete_genome. | Phage | Complete | G | NCBI |
| Enterobacteria_phage_RB32_complete_genome. | Phage | Complete | G | NCBI |
| Enterobacteria_phage_RB43_complete_genome. | Phage | Complete | G | NCBI |
| Enterobacteria_phage_RB49_complete_genome. | Phage | Complete | G | NCBI |
| Enterobacteria_phage_RB51_complete_genome. | Phage | Complete | G | NCBI |
| Enterobacteria_phage_RB69_complete_genome. | Phage | Complete | G | NCBI |
| Enterobacteria_phage_RTP_complete_genome. | Phage | Complete | G | NCBI |
| Enterobacteria_phage_Sf6_complete_genome. | Phage | Complete | G | NCBI |
| Enterobacteria_phage_SfV_complete_genome. | Phage | Complete | G | NCBI |
| Enterobacteria_phage_SP6_complete_genome. | Phage | Complete | G | NCBI |
| Enterobacteria_phage_SPC35_complete_genome. | Phage | Complete | G | NCBI |
| Enterobacteria_phage_SSL-2009a_complete_genome. | Phage | Complete | G | NCBI |
| Enterobacteria_phage_St-1_complete_genome. | Phage | Complete | G | NCBI |
| Enterobacteria_phage_ST104_complete_genome. | Phage | Complete | G | NCBI |
| Enterobacteria_phage_ST64T_complete_genome. | Phage | Complete | G | NCBI |
| Enterobacteria_phage_T1_complete_genome. | Phage | Complete | G | NCBI |
| Enterobacteria_phage_T3_complete_genome. | Phage | Complete | G | NCBI |
| Enterobacteria_phage_T4_complete_genome. | Phage | Complete | G | NCBI |
| Enterobacteria_phage_T5_complete_genome. | Phage | Complete | G | NCBI |
| Enterobacteria_phage_T7_complete_genome. | Phage | Complete | G | NCBI |
| Enterobacteria_phage_TLS_complete_genome. | Phage | Complete | G | NCBI |
| Enterobacteria_phage_vB_EcoM-VR7_complete_genome. | Phage | Complete | G | NCBI |
| Enterobacteria_phage_VT2-Sakai_complete_genome. | Phage | Complete | G | NCBI |
| Enterobacteria_phage_WA13_complete_genome. | Phage | Complete | G | NCBI |
| Enterobacteria_phage_WV8_complete_genome. | Phage | Complete | G | NCBI |
| Enterobacteria_phage_YYZ-2008_complete_prophage_genome. | Phage | Complete | G | NCBI |
| Enterobacterio_phage_MS2_complete_genome. | Phage | Complete | G | NCBI |
| Enterococcus_phage_EFAP-1_complete_genome. | Phage | Complete | G | NCBI |
| Enterococcus_phage_EFRM31_complete_genome. | Phage | Complete | G | NCBI |
| Enterococcus_phage_phiEf11_complete_genome. | Phage | Complete | G | NCBI |
| Enterococcus_phage_phiEF24C_complete_genome. | Phage | Complete | G | NCBI |
| Enterococcus_phage_phiFL1A_complete_genome. | Phage | Complete | G | NCBI |
| Enterococcus_phage_phiFL2A_complete_genome. | Phage | Complete | G | NCBI |
| Enterococcus_phage_phiFL3A_complete_genome. | Phage | Complete | G | NCBI |
| Enterococcus_phage_phiFL4A_complete_genome. | Phage | Complete | G | NCBI |
| Erwinia_amylovora_phage_Era103_complete_genome. | Phage | Complete | NG | NCBI |
| Erwinia_phage_phiEa104_complete_genome. | Phage | Complete | NG | NCBI |
| Erwinia_phage_phiEa21-4_complete_genome. | Phage | Complete | NG | NCBI |
| Erwinia_phage_phiEt88_complete_genome. | Phage | Complete | NG | NCBI |
| Escherichia_phage_D108_complete_genome. | Phage | Complete | G | NCBI |
| Escherichia_phage_phiV10_complete_genome. | Phage | Complete | G | NCBI |
| Escherichia_phage_rv5_complete_genome. | Phage | Complete | G | NCBI |
| Escherichia_phage_vB_EcoP_G7C_complete_genome. | Phage | Complete | G | NCBI |
| Flavobacterium_phage_11b_complete_genome. | Phage | Complete | - | NCBI |
| Geobacillus_phage_GBSV1_complete_genome. | Phage | Complete | - | NCBI |
| Geobacillus_virus_E2_complete_genome. | Phage | Complete | - | NCBI |
| Gordonia_phage_GTE2_complete_genome. | Phage | Complete | - | NCBI |
| Haemophilus_phage_HP1_complete_genome. | Phage | Complete | - | NCBI |
| Haemophilus_phage_HP2_complete_genome. | Phage | Complete | - | NCBI |
| Halomonas_phage_phiHAP-1_complete_genome. | Phage | Complete | - | NCBI |
| Iodobacteriophage_phiPLPE_complete_genome. | Phage | Complete | - | NCBI |
| Klebsiella_phage_K11_complete_genome. | Phage | Complete | G | NCBI |
| Klebsiella_phage_KP15_complete_genome. | Phage | Complete | G | NCBI |
| Klebsiella_phage_KP32_complete_genome. | Phage | Complete | G | NCBI |
| Klebsiella_phage_KP34_complete_genome. | Phage | Complete | G | NCBI |
| Klebsiella_phage_phiKO2_complete_genome. | Phage | Complete | G | NCBI |
| Kluyvera_phage_Kvp1_complete_genome. | Phage | Complete | - | NCBI |
| Lactobacillus_johnsonii_prophage_Lj771_complete_genome. | Phage | Complete | G | NCBI |
| Lactobacillus_phage_A2_complete_genome. | Phage | Complete | G | NCBI |
| Lactobacillus_phage_KC5a_complete_genome. | Phage | Complete | G | NCBI |
| Lactobacillus_phage_Lb338-1_complete_genome. | Phage | Complete | G | NCBI |
| Lactobacillus_phage_Lc-Nu_complete_genome. | Phage | Complete | G | NCBI |
| Lactobacillus_phage_LL-H_complete_genome. | Phage | Complete | G | NCBI |
| Lactobacillus_phage_LP65_complete_genome. | Phage | Complete | G | NCBI |
| Lactobacillus_phage_Lrm1_complete_genome. | Phage | Complete | G | NCBI |
| Lactobacillus_phage_Lv-1_complete_genome. | Phage | Complete | G | NCBI |
| Lactobacillus_phage_phiAT3_complete_genome. | Phage | Complete | G | NCBI |
| Lactobacillus_phage_phig1e_complete_genome. | Phage | Complete | G | NCBI |
| Lactobacillus_phage_phiJL-1_complete_genome. | Phage | Complete | G | NCBI |
| Lactobacillus_prophage_Lj928_complete_genome. | Phage | Complete | G | NCBI |
| Lactobacillus_prophage_Lj965_complete_genome. | Phage | Complete | G | NCBI |
| Lactobacillus_prophage_phiadh_complete_genome. | Phage | Complete | G | NCBI |
| Lactococcus_phage_1706_complete_genome. | Phage | Complete | G | NCBI |
| Lactococcus_phage_4268_complete_genome. | Phage | Complete | G | NCBI |
| Lactococcus_phage_712_complete_genome. | Phage | Complete | G | NCBI |
| Lactococcus_phage_949_complete_genome. | Phage | Complete | G | NCBI |
| Lactococcus_phage_asccphi28_complete_genome. | Phage | Complete | G | NCBI |
| Lactococcus_phage_bIBB29_complete_genome. | Phage | Complete | G | NCBI |
| Lactococcus_phage_bIL170_complete_genome. | Phage | Complete | G | NCBI |
| Lactococcus_phage_bIL67_complete_genome. | Phage | Complete | G | NCBI |
| Lactococcus_phage_BK5-T_complete_genome. | Phage | Complete | G | NCBI |
| Lactococcus_phage_c2_complete_genome. | Phage | Complete | G | NCBI |
| Lactococcus_phage_jj50_complete_genome. | Phage | Complete | G | NCBI |
| Lactococcus_phage_KSY1_complete_genome. | Phage | Complete | G | NCBI |
| Lactococcus_phage_P008_complete_genome. | Phage | Complete | G | NCBI |
| Lactococcus_phage_P087_complete_genome. | Phage | Complete | G | NCBI |
| Lactococcus_phage_phiLC3_complete_genome. | Phage | Complete | G | NCBI |
| Lactococcus_phage_Q54_complete_genome. | Phage | Complete | G | NCBI |
| Lactococcus_phage_r1t_complete_genome. | Phage | Complete | G | NCBI |
| Lactococcus_phage_sk1_complete_genome. | Phage | Complete | G | NCBI |
| Lactococcus_phage_TP901-1_complete_genome. | Phage | Complete | G | NCBI |
| Lactococcus_phage_Tuc2009_complete_genome. | Phage | Complete | G | NCBI |
| Lactococcus_phage_ul36_complete_genome. | Phage | Complete | G | NCBI |
| Lactococcus_prophage_bIL285_complete_genome. | Phage | Complete | G | NCBI |
| Lactococcus_prophage_bIL286_complete_genome. | Phage | Complete | G | NCBI |
| Lactococcus_prophage_bIL309_complete_genome. | Phage | Complete | G | NCBI |
| Lactococcus_prophage_bIL310_complete_genome. | Phage | Complete | G | NCBI |
| Lactococcus_prophage_bIL311_complete_genome. | Phage | Complete | G | NCBI |
| Lactococcus_prophage_bIL312_complete_genome. | Phage | Complete | G | NCBI |
| Listeria_phage_A006_complete_genome. | Phage | Complete | GA | NCBI |
| Listeria_phage_A118_complete_genome. | Phage | Complete | GA | NCBI |
| Listeria_phage_A500_complete_genome. | Phage | Complete | GA | NCBI |
| Listeria_phage_A511_complete_genome. | Phage | Complete | GA | NCBI |
| Listeria_phage_B025_complete_genome. | Phage | Complete | GA | NCBI |
| Listeria_phage_B054_complete_genome. | Phage | Complete | GA | NCBI |
| Listeria_phage_P35_complete_genome. | Phage | Complete | GA | NCBI |
| Listeria_phage_P40_complete_genome. | Phage | Complete | GA | NCBI |
| Listonella_phage_phiHSIC_complete_genome. | Phage | Complete | - | NCBI |
| Mannheimia_phage_phiMHaA1_complete_genome. | Phage | Complete | - | NCBI |
| Microbacterium_phage_Min1_complete_genome. | Phage | Complete | - | NCBI |
| Microcystis_phage_Ma-LMM01_complete_genome. | Phage | Complete | - | NCBI |
| Morganella_phage_MmP1_complete_genome. | Phage | Complete | - | NCBI |
| Mycobacterium_phage_244_complete_genome. | Phage | Complete | NG | NCBI |
| Mycobacterium_phage_Adjutor_complete_genome. | Phage | Complete | NG | NCBI |
| Mycobacterium_phage_Angel_complete_genome. | Phage | Complete | NG | NCBI |
| Mycobacterium_phage_Angelica_complete_genome. | Phage | Complete | NG | NCBI |
| Mycobacterium_phage_Ardmore_complete_genome. | Phage | Complete | NG | NCBI |
| Mycobacterium_phage_Barnyard_complete_genome. | Phage | Complete | NG | NCBI |
| Mycobacterium_phage_Bethlehem_complete_genome. | Phage | Complete | NG | NCBI |
| Mycobacterium_phage_Boomer_complete_genome. | Phage | Complete | NG | NCBI |
| Mycobacterium_phage_BPs_complete_genome. | Phage | Complete | NG | NCBI |
| Mycobacterium_phage_Brujita_complete_genome. | Phage | Complete | NG | NCBI |
| Mycobacterium_phage_Butterscotch_complete_genome. | Phage | Complete | NG | NCBI |
| Mycobacterium_phage_Bxb1_complete_genome. | Phage | Complete | NG | NCBI |
| Mycobacterium_phage_Bxz1_complete_genome. | Phage | Complete | NG | NCBI |
| Mycobacterium_phage_Bxz2_complete_genome. | Phage | Complete | NG | NCBI |
| Mycobacterium_phage_Cali_complete_genome. | Phage | Complete | NG | NCBI |
| Mycobacterium_phage_Catera_complete_genome. | Phage | Complete | NG | NCBI |
| Mycobacterium_phage_Chah_complete_genome. | Phage | Complete | NG | NCBI |
| Mycobacterium_phage_Che12_complete_genome. | Phage | Complete | NG | NCBI |
| Mycobacterium_phage_Che8_complete_genome. | Phage | Complete | NG | NCBI |
| Mycobacterium_phage_Che9c_complete_genome. | Phage | Complete | NG | NCBI |
| Mycobacterium_phage_Che9d_complete_genome. | Phage | Complete | NG | NCBI |
| Mycobacterium_phage_Cjw1_complete_genome. | Phage | Complete | NG | NCBI |
| Mycobacterium_phage_Cooper_complete_genome. | Phage | Complete | NG | NCBI |
| Mycobacterium_phage_Corndog_complete_genome. | Phage | Complete | NG | NCBI |
| Mycobacterium_phage_CrimD_complete_genome. | Phage | Complete | NG | NCBI |
| Mycobacterium_phage_D29_complete_genome. | Phage | Complete | NG | NCBI |
| Mycobacterium_phage_DD5_complete_genome. | Phage | Complete | NG | NCBI |
| Mycobacterium_phage_ET08_complete_genome. | Phage | Complete | NG | NCBI |
| Mycobacterium_phage_Faith1_complete_genome. | Phage | Complete | NG | NCBI |
| Mycobacterium_phage_Fruitloop_complete_genome. | Phage | Complete | NG | NCBI |
| Mycobacterium_phage_Giles_complete_genome. | Phage | Complete | NG | NCBI |
| Mycobacterium_phage_Gumball_complete_genome. | Phage | Complete | NG | NCBI |
| Mycobacterium_phage_Halo_complete_genome. | Phage | Complete | NG | NCBI |
| Mycobacterium_phage_Jasper_complete_genome. | Phage | Complete | NG | NCBI |
| Mycobacterium_phage_KBG_complete_genome. | Phage | Complete | NG | NCBI |
| Mycobacterium_phage_Konstantine_complete_genome. | Phage | Complete | NG | NCBI |
| Mycobacterium_phage_Kostya_complete_genome. | Phage | Complete | NG | NCBI |
| Mycobacterium_phage_L5_complete_genome. | Phage | Complete | NG | NCBI |
| Mycobacterium_phage_LeBron_complete_genome. | Phage | Complete | NG | NCBI |
| Mycobacterium_phage_Llij_complete_genome. | Phage | Complete | NG | NCBI |
| Mycobacterium_phage_Lockley_complete_genome. | Phage | Complete | NG | NCBI |
| Mycobacterium_phage_Myrna_complete_genome. | Phage | Complete | NG | NCBI |
| Mycobacterium_phage_Nigel_complete_genome. | Phage | Complete | NG | NCBI |
| Mycobacterium_phage_Omega_complete_genome. | Phage | Complete | NG | NCBI |
| Mycobacterium_phage_Orion_complete_genome. | Phage | Complete | NG | NCBI |
| Mycobacterium_phage_Pacc40_complete_genome. | Phage | Complete | NG | NCBI |
| Mycobacterium_phage_PBI1_complete_genome. | Phage | Complete | NG | NCBI |
| Mycobacterium_phage_Peaches_complete_genome. | Phage | Complete | NG | NCBI |
| Mycobacterium_phage_PG1_complete_genome. | Phage | Complete | NG | NCBI |
| Mycobacterium_phage_Phaedrus_complete_genome. | Phage | Complete | NG | NCBI |
| Mycobacterium_phage_Phlyer_complete_genome. | Phage | Complete | NG | NCBI |
| Mycobacterium_phage_Pipefish_complete_genome. | Phage | Complete | NG | NCBI |
| Mycobacterium_phage_PLot_complete_genome. | Phage | Complete | NG | NCBI |
| Mycobacterium_phage_PMC_complete_genome. | Phage | Complete | NG | NCBI |
| Mycobacterium_phage_Porky_complete_genome. | Phage | Complete | NG | NCBI |
| Mycobacterium_phage_Predator_complete_genome. | Phage | Complete | NG | NCBI |
| Mycobacterium_phage_Pukovnik_complete_genome. | Phage | Complete | NG | NCBI |
| Mycobacterium_phage_Qyrzula_complete_genome. | Phage | Complete | NG | NCBI |
| Mycobacterium_phage_Ramsey_complete_genome. | Phage | Complete | NG | NCBI |
| Mycobacterium_phage_Rizal_complete_genome. | Phage | Complete | NG | NCBI |
| Mycobacterium_phage_Rosebush_complete_genome. | Phage | Complete | NG | NCBI |
| Mycobacterium_phage_ScottMcG_complete_genome. | Phage | Complete | NG | NCBI |
| Mycobacterium_phage_Solon_complete_genome. | Phage | Complete | NG | NCBI |
| Mycobacterium_phage_Spud_complete_genome. | Phage | Complete | NG | NCBI |
| Mycobacterium_phage_TM4_complete_genome. | Phage | Complete | NG | NCBI |
| Mycobacterium_phage_Troll4_complete_genome. | Phage | Complete | NG | NCBI |
| Mycobacterium_phage_Tweety_complete_genome. | Phage | Complete | NG | NCBI |
| Mycobacterium_phage_U2_complete_genome. | Phage | Complete | NG | NCBI |
| Mycobacterium_phage_Wee_complete_genome. | Phage | Complete | NG | NCBI |
| Mycobacterium_phage_Wildcat_complete_genome. | Phage | Complete | NG | NCBI |
| Mycoplasma_phage_MAV1_complete_genome. | Phage | Complete | - | NCBI |
| Mycoplasma_phage_P1_complete_genome. | Phage | Complete | - | NCBI |
| Mycoplasma_phage_phiMFV1_complete_genome. | Phage | Complete | - | NCBI |
| Myxococcus_phage_Mx8_complete_genome. | Phage | Complete | - | NCBI |
| Pantoea_phage_LIMEzero_complete_genome. | Phage | Complete | - | NCBI |
| Pasteurella_phage_F108_complete_genome. | Phage | Complete | - | NCBI |
| Phage_cdtI_complete_genome. | Phage | Complete | - | NCBI |
| Phage_Gifsy-1_complete_genome. | Phage | Complete | - | NCBI |
| Phage_Gifsy-2_complete_genome. | Phage | Complete | - | NCBI |
| Phage_phiJL001_complete_genome. | Phage | Complete | - | NCBI |
| Phormidium_phage_Pf-WMP3_complete_genome. | Phage | Complete | - | NCBI |
| Phormidium_phage_Pf-WMP4_complete_genome. | Phage | Complete | - | NCBI |
| Prochlorococcus_phage_P-HM1_complete_genome. | Phage | Complete | NG | NCBI |
| Prochlorococcus_phage_P-HM2_complete_genome. | Phage | Complete | NG | NCBI |
| Prochlorococcus_phage_P-RSM4_complete_genome. | Phage | Complete | NG | NCBI |
| Prochlorococcus_phage_P-SSM2_complete_genome. | Phage | Complete | NG | NCBI |
| Prochlorococcus_phage_P-SSM4_complete_genome. | Phage | Complete | NG | NCBI |
| Prochlorococcus_phage_P-SSM7_complete_genome. | Phage | Complete | NG | NCBI |
| Prochlorococcus_phage_P-SSP7_complete_genome. | Phage | Complete | NG | NCBI |
| Prochlorococcus_phage_Syn1_complete_genome. | Phage | Complete | NG | NCBI |
| Prochlorococcus_phage_Syn33_complete_genome. | Phage | Complete | NG | NCBI |
| Propionibacterium_phage_B5_complete_genome. | Phage | Complete | NG | NCBI |
| Propionibacterium_phage_PA6_complete_genome. | Phage | Complete | NG | NCBI |
| Propionibacterium_phage_PAD20_endogenous_virus_complete_genome. | Phage | Complete | NG | NCBI |
| Propionibacterium_phage_PAS50_endogenous_virus_complete_genome. | Phage | Complete | NG | NCBI |
| Pseudoalteromonas_phage_H105_1_complete_genome. | Phage | Complete | - | NCBI |
| Pseudoalteromonas_phage_PM2_complete_genome. | Phage | Complete | - | NCBI |
| Pseudomonas_phage_119X_complete_genome. | Phage | Complete | NG | NCBI |
| Pseudomonas_phage_14-1_complete_genome. | Phage | Complete | NG | NCBI |
| Pseudomonas_phage_201phi2-1_complete_genome. | Phage | Complete | NG | NCBI |
| Pseudomonas_phage_73_complete_genome. | Phage | Complete | NG | NCBI |
| Pseudomonas_phage_B3_complete_genome. | Phage | Complete | NG | NCBI |
| Pseudomonas_phage_D3_complete_genome. | Phage | Complete | NG | NCBI |
| Pseudomonas_phage_D3112_complete_genome. | Phage | Complete | NG | NCBI |
| Pseudomonas_phage_DMS3_complete_genome. | Phage | Complete | NG | NCBI |
| Pseudomonas_phage_EL_complete_genome. | Phage | Complete | NG | NCBI |
| Pseudomonas_phage_F10_complete_genome. | Phage | Complete | NG | NCBI |
| Pseudomonas_phage_F116_complete_genome. | Phage | Complete | NG | NCBI |
| Pseudomonas_phage_F8_complete_genome. | Phage | Complete | NG | NCBI |
| Pseudomonas_phage_gh-1_complete_genome. | Phage | Complete | NG | NCBI |
| Pseudomonas_phage_KPP10_complete_genome. | Phage | Complete | NG | NCBI |
| Pseudomonas_phage_LBL3_complete_genome. | Phage | Complete | NG | NCBI |
| Pseudomonas_phage_LIT1_complete_genome. | Phage | Complete | NG | NCBI |
| Pseudomonas_phage_LKD16_complete_genome. | Phage | Complete | NG | NCBI |
| Pseudomonas_phage_LMA2_complete_genome. | Phage | Complete | NG | NCBI |
| Pseudomonas_phage_LUZ19_complete_genome. | Phage | Complete | NG | NCBI |
| Pseudomonas_phage_LUZ24_complete_genome. | Phage | Complete | NG | NCBI |
| Pseudomonas_phage_LUZ7_complete_genome. | Phage | Complete | NG | NCBI |
| Pseudomonas_phage_M6_complete_genome. | Phage | Complete | NG | NCBI |
| Pseudomonas_phage_MP22_complete_genome. | Phage | Complete | NG | NCBI |
| Pseudomonas_phage_MP29_complete_genome. | Phage | Complete | NG | NCBI |
| Pseudomonas_phage_MP38_complete_genome. | Phage | Complete | NG | NCBI |
| Pseudomonas_phage_PA11_complete_genome. | Phage | Complete | NG | NCBI |
| Pseudomonas_phage_PAJU2_complete_genome. | Phage | Complete | NG | NCBI |
| Pseudomonas_phage_PAK_P1_complete_genome. | Phage | Complete | NG | NCBI |
| Pseudomonas_phage_PaP2_complete_genome. | Phage | Complete | NG | NCBI |
| Pseudomonas_phage_PaP3_complete_genome. | Phage | Complete | NG | NCBI |
| Pseudomonas_phage_PB1_complete_genome. | Phage | Complete | NG | NCBI |
| Pseudomonas_phage_Pf1_complete_genome. | Phage | Complete | NG | NCBI |
| Pseudomonas_phage_Pf3_complete_genome. | Phage | Complete | NG | NCBI |
| Pseudomonas_phage_phi-2_complete_genome. | Phage | Complete | NG | NCBI |
| Pseudomonas_phage_phi13_segment_L_complete_genome. | Phage | Complete | NG | NCBI |
| Pseudomonas_phage_phi15_complete_genome. | Phage | Complete | NG | NCBI |
| Pseudomonas_phage_phi2954_segment_L_complete_genome. | Phage | Complete | NG | NCBI |
| Pseudomonas_phage_phi2954_segment_M_complete_genome. | Phage | Complete | NG | NCBI |
| Pseudomonas_phage_phi2954_segment_S_complete_genome. | Phage | Complete | NG | NCBI |
| Pseudomonas_phage_phi8_segment_L_complete_sequence. | Phage | Complete | NG | NCBI |
| Pseudomonas_phage_phi8_segment_S_complete_sequence. | Phage | Complete | NG | NCBI |
| Pseudomonas_phage_phiCTX_complete_genome. | Phage | Complete | NG | NCBI |
| Pseudomonas_phage_phiIBB-PF7A_complete_genome. | Phage | Complete | NG | NCBI |
| Pseudomonas_phage_phikF77_complete_genome. | Phage | Complete | NG | NCBI |
| Pseudomonas_phage_phiKMV_complete_genome. | Phage | Complete | NG | NCBI |
| Pseudomonas_phage_phiKZ_complete_genome. | Phage | Complete | NG | NCBI |
| Pseudomonas_phage_PP7_complete_genome. | Phage | Complete | NG | NCBI |
| Pseudomonas_phage_PRR1_complete_genome. | Phage | Complete | NG | NCBI |
| Pseudomonas_phage_PT2_complete_genome. | Phage | Complete | NG | NCBI |
| Pseudomonas_phage_PT5_complete_genome. | Phage | Complete | NG | NCBI |
| Pseudomonas_phage_SN_complete_genome. | Phage | Complete | NG | NCBI |
| Pseudomonas_phage_YuA_complete_genome. | Phage | Complete | NG | NCBI |
| Ralstonia_phage_p12J_complete_genome. | Phage | Complete | NG | NCBI |
| Ralstonia_phage_PE226_complete_genome. | Phage | Complete | NG | NCBI |
| Ralstonia_phage_phiRSA1_complete_genome. | Phage | Complete | NG | NCBI |
| Ralstonia_phage_RSB1_complete_genome. | Phage | Complete | NG | NCBI |
| Ralstonia_phage_RSL1_complete_genome. | Phage | Complete | NG | NCBI |
| Ralstonia_phage_RSM1_complete_genome. | Phage | Complete | NG | NCBI |
| Ralstonia_phage_RSM3_complete_genome. | Phage | Complete | NG | NCBI |
| Ralstonia_phage_RSS1_complete_genome. | Phage | Complete | NG | NCBI |
| Rhizobium_phage_16-3_complete_genome. | Phage | Complete | - | NCBI |
| Rhodothermus_phage_RM378_complete_genome. | Phage | Complete | - | NCBI |
| Roseobacter_phage_RDJL_Phi_1_complete_genome. | Phage | Complete | - | NCBI |
| Roseobacter_phage_SIO1_complete_genome. | Phage | Complete | - | NCBI |
| Roseophage_DSS3P2_complete_genome. | Phage | Complete | - | NCBI |
| Roseophage_EE36P1_complete_genome. | Phage | Complete | - | NCBI |
| Salmonella_enterica_bacteriophage_SE1_complete_genome. | Phage | Complete | GA | NCBI |
| Salmonella_phage_7-11_complete_genome. | Phage | Complete | GA | NCBI |
| Salmonella_phage_c341_complete_genome. | Phage | Complete | GA | NCBI |
| Salmonella_phage_E1_complete_genome. | Phage | Complete | GA | NCBI |
| Salmonella_phage_epsilon34_complete_genome. | Phage | Complete | GA | NCBI |
| Salmonella_phage_Fels-1_complete_genome. | Phage | Complete | GA | NCBI |
| Salmonella_phage_phiSG-JL2_complete_genome. | Phage | Complete | GA | NCBI |
| Salmonella_phage_SETP3_complete_genome. | Phage | Complete | GA | NCBI |
| Salmonella_phage_SS3e_complete_genome. | Phage | Complete | GA | NCBI |
| Salmonella_phage_ST160_complete_genome. | Phage | Complete | GA | NCBI |
| Salmonella_phage_ST64B_complete_genome. | Phage | Complete | GA | NCBI |
| Salmonella_phage_Vi01_complete_genome. | Phage | Complete | GA | NCBI |
| Salmonella_phage_Vi06_complete_genome. | Phage | Complete | GA | NCBI |
| Shigella_phage_phiSboM-AG3_complete_genome. | Phage | Complete | GA | NCBI |
| Shigella_phage_Shfl1_complete_genome. | Phage | Complete | GA | NCBI |
| Shigella_phage_Shfl2_complete_genome. | Phage | Complete | GA | NCBI |
| Shigella_phage_SP18_complete_genome. | Phage | Complete | GA | NCBI |
| Sinorhizobium_phage_PBC5_complete_genome. | Phage | Complete | - | NCBI |
| Sodalis_phage_phiSG1_complete_genome. | Phage | Complete | - | NCBI |
| Sodalis_phage_SO-1_complete_genome. | Phage | Complete | - | NCBI |
| Spiroplasma_kunkelii_virus_SkV1_CR2-3x_complete_genome. | Phage | Complete | - | NCBI |
| Spiroplasma_phage_1-C74_complete_genome. | Phage | Complete | NG | NCBI |
| Spiroplasma_phage_1-R8A2B_complete_genome. | Phage | Complete | NG | NCBI |
| Spiroplasma_phage_4_complete_genome. | Phage | Complete | NG | NCBI |
| Spiroplasma_phage_SVTS2_complete_genome. | Phage | Complete | NG | NCBI |
| Staphylococcus_phage_11_complete_genome. | Phage | Complete | G | NCBI |
| Staphylococcus_phage_187_complete_genome. | Phage | Complete | G | NCBI |
| Staphylococcus_phage_2638A_complete_genome. | Phage | Complete | G | NCBI |
| Staphylococcus_phage_29_complete_genome. | Phage | Complete | G | NCBI |
| Staphylococcus_phage_37_complete_genome. | Phage | Complete | G | NCBI |
| Staphylococcus_phage_3A_complete_genome. | Phage | Complete | G | NCBI |
| Staphylococcus_phage_42e_complete_genome. | Phage | Complete | G | NCBI |
| Staphylococcus_phage_44AHJD_complete_genome. | Phage | Complete | G | NCBI |
| Staphylococcus_phage_47_complete_genome. | Phage | Complete | G | NCBI |
| Staphylococcus_phage_52A_complete_genome. | Phage | Complete | G | NCBI |
| Staphylococcus_phage_53_complete_genome. | Phage | Complete | G | NCBI |
| Staphylococcus_phage_55_complete_genome. | Phage | Complete | G | NCBI |
| Staphylococcus_phage_66_complete_genome. | Phage | Complete | G | NCBI |
| Staphylococcus_phage_69_complete_genome. | Phage | Complete | G | NCBI |
| Staphylococcus_phage_71_complete_genome. | Phage | Complete | G | NCBI |
| Staphylococcus_phage_77_complete_genome. | Phage | Complete | G | NCBI |
| Staphylococcus_phage_80alpha_complete_genome. | Phage | Complete | G | NCBI |
| Staphylococcus_phage_85_complete_genome. | Phage | Complete | G | NCBI |
| Staphylococcus_phage_88_complete_genome. | Phage | Complete | G | NCBI |
| Staphylococcus_phage_92_complete_genome. | Phage | Complete | G | NCBI |
| Staphylococcus_phage_96_complete_genome. | Phage | Complete | G | NCBI |
| Staphylococcus_phage_CNPH82_complete_genome. | Phage | Complete | G | NCBI |
| Staphylococcus_phage_EW_complete_genome. | Phage | Complete | G | NCBI |
| Staphylococcus_phage_G1_complete_genome. | Phage | Complete | G | NCBI |
| Staphylococcus_phage_K_complete_genome. | Phage | Complete | G | NCBI |
| Staphylococcus_phage_P954_complete_genome. | Phage | Complete | G | NCBI |
| Staphylococcus_phage_PH15_complete_genome. | Phage | Complete | G | NCBI |
| Staphylococcus_phage_phi2958PVL_complete_genome. | Phage | Complete | G | NCBI |
| Staphylococcus_phage_phiETA_complete_genome. | Phage | Complete | G | NCBI |
| Staphylococcus_phage_phiETA2_complete_genome. | Phage | Complete | G | NCBI |
| Staphylococcus_phage_phiETA3_complete_genome. | Phage | Complete | G | NCBI |
| Staphylococcus_phage_phiMR11_complete_genome. | Phage | Complete | G | NCBI |
| Staphylococcus_phage_phiMR25_complete_genome. | Phage | Complete | G | NCBI |
| Staphylococcus_phage_phiNM1_complete_genome. | Phage | Complete | G | NCBI |
| Staphylococcus_phage_phiNM3_complete_genome. | Phage | Complete | G | NCBI |
| Staphylococcus_phage_phiP68_complete_genome. | Phage | Complete | G | NCBI |
| Staphylococcus_phage_phiPVL-CN125_complete_genome. | Phage | Complete | G | NCBI |
| Staphylococcus_phage_phiPVL108_complete_genome. | Phage | Complete | G | NCBI |
| Staphylococcus_phage_phiSauS-IPLA35_complete_genome. | Phage | Complete | G | NCBI |
| Staphylococcus_phage_phiSauS-IPLA88_complete_genome. | Phage | Complete | G | NCBI |
| Staphylococcus_phage_phiSLT_complete_genome. | Phage | Complete | G | NCBI |
| Staphylococcus_phage_PT1028_complete_genome. | Phage | Complete | G | NCBI |
| Staphylococcus_phage_ROSA_complete_genome. | Phage | Complete | G | NCBI |
| Staphylococcus_phage_SAP-2_complete_genome. | Phage | Complete | G | NCBI |
| Staphylococcus_phage_SAP-26_complete_genome. | Phage | Complete | G | NCBI |
| Staphylococcus_phage_Twort_complete_genome. | Phage | Complete | G | NCBI |
| Staphylococcus_phage_X2_complete_genome. | Phage | Complete | G | NCBI |
| Staphylococcus_prophage_phi_12_complete_genome. | Phage | Complete | G | NCBI |
| Staphylococcus_prophage_phi_13_complete_genome. | Phage | Complete | G | NCBI |
| Staphylococcus_prophage_phiN315_complete_genome. | Phage | Complete | G | NCBI |
| Staphylococcus_prophage_phiPV83_complete_genome. | Phage | Complete | G | NCBI |
| Staphylococcus_prophage_PVL_complete_genome. | Phage | Complete | G | NCBI |
| Staphylococcus_prophage_tp310-1_complete_genome. | Phage | Complete | G | NCBI |
| Staphylococcus_prophage_tp310-2_complete_genome. | Phage | Complete | G | NCBI |
| Staphylococcus_prophage_tp310-3_complete_genome. | Phage | Complete | G | NCBI |
| Stenotrophomonas_phage_phiSMA9_complete_genome. | Phage | Complete | - | NCBI |
| Stenotrophomonas_phage_S1_complete_genome. | Phage | Complete | - | NCBI |
| Streptococcus_phage_2972_complete_genome. | Phage | Complete | G | NCBI |
| Streptococcus_phage_5093_complete_genome. | Phage | Complete | G | NCBI |
| Streptococcus_phage_7201_complete_genome. | Phage | Complete | G | NCBI |
| Streptococcus_phage_858_complete_genome. | Phage | Complete | G | NCBI |
| Streptococcus_phage_Abc2_complete_genome. | Phage | Complete | G | NCBI |
| Streptococcus_phage_ALQ13.2_complete_genome. | Phage | Complete | G | NCBI |
| Streptococcus_phage_C1_complete_genome. | Phage | Complete | G | NCBI |
| Streptococcus_phage_Cp-1_complete_genome. | Phage | Complete | G | NCBI |
| Streptococcus_phage_Dp-1_complete_genome. | Phage | Complete | G | NCBI |
| Streptococcus_phage_DT1_complete_genome. | Phage | Complete | G | NCBI |
| Streptococcus_phage_M102_complete_genome. | Phage | Complete | G | NCBI |
| Streptococcus_phage_O1205_complete_genome. | Phage | Complete | G | NCBI |
| Streptococcus_phage_P9_complete_genome. | Phage | Complete | G | NCBI |
| Streptococcus_phage_PH10_complete_genome. | Phage | Complete | G | NCBI |
| Streptococcus_phage_PH15_complete_genome. | Phage | Complete | G | NCBI |
| Streptococcus_phage_phi3396_complete_genome. | Phage | Complete | G | NCBI |
| Streptococcus_phage_Sfi11_complete_genome. | Phage | Complete | G | NCBI |
| Streptococcus_phage_Sfi19_complete_genome. | Phage | Complete | G | NCBI |
| Streptococcus_phage_Sfi21_complete_genome. | Phage | Complete | G | NCBI |
| Streptococcus_phage_SM1_complete_genome. | Phage | Complete | G | NCBI |
| Streptococcus_phage_SMP_complete_genome. | Phage | Complete | G | NCBI |
| Streptococcus_prophage_315.1_complete_genome. | Phage | Complete | G | NCBI |
| Streptococcus_prophage_315.2_complete_genome. | Phage | Complete | G | NCBI |
| Streptococcus_prophage_315.4_complete_genome. | Phage | Complete | G | NCBI |
| Streptococcus_prophage_315.5_complete_genome. | Phage | Complete | G | NCBI |
| Streptococcus_prophage_315.6_complete_genome. | Phage | Complete | G | NCBI |
| Streptococcus_prophage_EJ-1_complete_genome. | Phage | Complete | G | NCBI |
| Streptococcus_prophage_MM1_complete_genome. | Phage | Complete | G | NCBI |
| Streptococcus_pyogenes_phage_315.3_complete_genome. | Phage | Complete | G | NCBI |
| Streptomyces_phage_mu1_6_complete_genome. | Phage | Complete | NG | NCBI |
| Streptomyces_phage_phiBT1_complete_genome. | Phage | Complete | NG | NCBI |
| Streptomyces_phage_phiC31_complete_genome. | Phage | Complete | NG | NCBI |
| Streptomyces_phage_phiSASD1_complete_genome. | Phage | Complete | NG | NCBI |
| Streptomyces_phage_VWB_complete_genome. | Phage | Complete | NG | NCBI |
| Stx1_converting_phage_complete_genome. | Phage | Complete | - | NCBI |
| Stx2_converting_phage_I_complete_genome. | Phage | Complete | - | NCBI |
| Stx2_converting_phage_II_complete_genome. | Phage | Complete | - | NCBI |
| Stx2-converting_phage_1717_complete_prophage_genome. | Phage | Complete | - | NCBI |
| Stx2-converting_phage_86_complete_genome. | Phage | Complete | - | NCBI |
| Synechococcus_phage_P60_complete_genome. | Phage | Complete | NG | NCBI |
| Synechococcus_phage_S-CBS2_complete_genome. | Phage | Complete | NG | NCBI |
| Synechococcus_phage_S-CBS3_complete_genome. | Phage | Complete | NG | NCBI |
| Synechococcus_phage_S-CRM01_complete_genome. | Phage | Complete | NG | NCBI |
| Synechococcus_phage_S-PM2_complete_genome. | Phage | Complete | NG | NCBI |
| Synechococcus_phage_S-RSM4_complete_genome. | Phage | Complete | NG | NCBI |
| Synechococcus_phage_S-ShM2_complete_genome. | Phage | Complete | NG | NCBI |
| Synechococcus_phage_S-SM1_complete_genome. | Phage | Complete | NG | NCBI |
| Synechococcus_phage_S-SM2_complete_genome. | Phage | Complete | NG | NCBI |
| Synechococcus_phage_S-SSM5_complete_genome. | Phage | Complete | NG | NCBI |
| Synechococcus_phage_S-SSM7_complete_genome. | Phage | Complete | NG | NCBI |
| Synechococcus_phage_Syn19_complete_genome. | Phage | Complete | NG | NCBI |
| Synechococcus_phage_syn9_complete_genome. | Phage | Complete | NG | NCBI |
| Temperate_phage_phiNIH1.1_complete_genome. | Phage | Complete | - | NCBI |
| Thalassomonas_phage_BA3_complete_genome. | Phage | Complete | - | NCBI |
| Thermus_phage_IN93_complete_genome. | Phage | Complete | NG | NCBI |
| Thermus_phage_P23-45_complete_genome. | Phage | Complete | NG | NCBI |
| Thermus_phage_P23-77_complete_genome. | Phage | Complete | NG | NCBI |
| Thermus_phage_P74-26_complete_genome. | Phage | Complete | NG | NCBI |
| Thermus_phage_phiYS40_complete_genome. | Phage | Complete | NG | NCBI |
| Thermus_phage_TMA_complete_genome. | Phage | Complete | NG | NCBI |
| Tsukamurella_phage_TPA2_complete_genome. | Phage | Complete | - | NCBI |
| Vibrio_phage_CTX_chromosome_I_complete_genome. | Phage | Complete | GA | NCBI |
| Vibrio_phage_fs1_complete_genome. | Phage | Complete | GA | NCBI |
| Vibrio_phage_fs2_complete_genome. | Phage | Complete | GA | NCBI |
| Vibrio_phage_ICP1_complete_genome. | Phage | Complete | GA | NCBI |
| Vibrio_phage_ICP2_complete_genome. | Phage | Complete | GA | NCBI |
| Vibrio_phage_ICP3_complete_genome. | Phage | Complete | GA | NCBI |
| Vibrio_phage_K139_complete_genome. | Phage | Complete | GA | NCBI |
| Vibrio_phage_kappa_complete_genome. | Phage | Complete | GA | NCBI |
| Vibrio_phage_KSF-1phi_complete_genome. | Phage | Complete | GA | NCBI |
| Vibrio_phage_KVP40_complete_genome. | Phage | Complete | GA | NCBI |
| Vibrio_phage_N4_complete_genome. | Phage | Complete | GA | NCBI |
| Vibrio_phage_VEJphi_complete_genome. | Phage | Complete | GA | NCBI |
| Vibrio_phage_Vf12_complete_genome. | Phage | Complete | GA | NCBI |
| Vibrio_phage_Vf33_complete_genome. | Phage | Complete | GA | NCBI |
| Vibrio_phage_VfO3K6_complete_genome. | Phage | Complete | GA | NCBI |
| Vibrio_phage_VfO4K68_complete_genome. | Phage | Complete | GA | NCBI |
| Vibrio_phage_VGJphi_complete_genome. | Phage | Complete | GA | NCBI |
| Vibrio_phage_VHML_complete_genome. | Phage | Complete | GA | NCBI |
| Vibrio_phage_VP2_complete_genome. | Phage | Complete | GA | NCBI |
| Vibrio_phage_VP5_complete_genome. | Phage | Complete | GA | NCBI |
| Vibrio_phage_VP882_complete_genome. | Phage | Complete | GA | NCBI |
| Vibrio_phage_VP93_complete_genome. | Phage | Complete | GA | NCBI |
| Vibrio_phage_VpV262_complete_genome. | Phage | Complete | GA | NCBI |
| Vibrio_phage_VSK_complete_genome. | Phage | Complete | GA | NCBI |
| Vibriophage_VP4_complete_genome. | Phage | Complete | GA | NCBI |
| Xanthomonas_phage_Cf1c_complete_genome. | Phage | Complete | NG | NCBI |
| Xanthomonas_phage_OP1_complete_genome. | Phage | Complete | NG | NCBI |
| Xanthomonas_phage_OP2_complete_genome. | Phage | Complete | NG | NCBI |
| Xanthomonas_phage_phiL7_complete_genome. | Phage | Complete | NG | NCBI |
| Xanthomonas_phage_Xop411_complete_genome. | Phage | Complete | NG | NCBI |
| Xanthomonas_phage_Xp10_complete_genome. | Phage | Complete | NG | NCBI |
| Xanthomonas_phage_Xp15_complete_genome. | Phage | Complete | NG | NCBI |
| Xylella_phage_Xfas53_complete_genome. | Phage | Complete | - | NCBI |
| Yersinia_pestis_phage_phiA1122_complete_genome. | Phage | Complete | GA | NCBI |
| Yersinia_phage_Berlin_complete_genome. | Phage | Complete | GA | NCBI |
| Yersinia_phage_L-413C_complete_genome. | Phage | Complete | GA | NCBI |
| Yersinia_phage_phiYeO3-12_complete_genome. | Phage | Complete | GA | NCBI |
| Yersinia_phage_PY54_complete_genome. | Phage | Complete | GA | NCBI |
| Yersinia_phage_Yepe2_complete_genome. | Phage | Complete | GA | NCBI |
| F1M CONT 237 | Viral Metagenome fragment | N/A | G | NCBI SRA |
| F1M CONT 240 | Viral Metagenome fragment | N/A | G | NCBI SRA |
| F1M CONT 241 | Viral Metagenome fragment | N/A | G | NCBI SRA |
| F1M CONT 243 | Viral Metagenome fragment | N/A | G | NCBI SRA |
| F1M CONT 245 | Viral Metagenome fragment | N/A | G | NCBI SRA |
| F1M CONT 247 | Viral Metagenome fragment | N/A | G | NCBI SRA |
| F1M CONT 248 | Viral Metagenome fragment | N/A | G | NCBI SRA |
| F1M CONT 249 | Viral Metagenome fragment | N/A | G | NCBI SRA |
| F1M CONT 250 | Viral Metagenome fragment | N/A | G | NCBI SRA |
| F1M CONT 251 | Viral Metagenome fragment | N/A | G | NCBI SRA |
| F1M CONT 252 | Viral Metagenome fragment | N/A | G | NCBI SRA |
| F1M CONT 253 | Viral Metagenome fragment | N/A | G | NCBI SRA |
| F1M CONT 254 | Viral Metagenome fragment | N/A | G | NCBI SRA |
| F1M CONT 255 | Viral Metagenome fragment | N/A | G | NCBI SRA |
| F1M CONT 355 | Viral Metagenome fragment | N/A | G | NCBI SRA |
| F1M CONT 364 | Viral Metagenome fragment | N/A | G | NCBI SRA |
| F1M CONT 372 | Viral Metagenome fragment | N/A | G | NCBI SRA |
| F1M CONT 373 | Viral Metagenome fragment | N/A | G | NCBI SRA |
| F1M CONT 374 | Viral Metagenome fragment | N/A | G | NCBI SRA |
| F1M CONT 376 | Viral Metagenome fragment | N/A | G | NCBI SRA |
| F1M CONT 380 | Viral Metagenome fragment | N/A | G | NCBI SRA |
| F1M CONT 381 | Viral Metagenome fragment | N/A | G | NCBI SRA |
| F1M CONT 382 | Viral Metagenome fragment | N/A | G | NCBI SRA |
| F1M CONT 384 | Viral Metagenome fragment | N/A | G | NCBI SRA |
| F1M CONT 385 | Viral Metagenome fragment | N/A | G | NCBI SRA |
| F1M CONT 386 | Viral Metagenome fragment | N/A | G | NCBI SRA |
| F1M CONT 387 | Viral Metagenome fragment | N/A | G | NCBI SRA |
| F1M CONT 388 | Viral Metagenome fragment | N/A | G | NCBI SRA |
| F1T1 CONT 15 | Viral Metagenome fragment | N/A | G | NCBI SRA |
| F1T1 CONT 16 | Viral Metagenome fragment | N/A | G | NCBI SRA |
| F1T1 CONT 308 | Viral Metagenome fragment | N/A | G | NCBI SRA |
| F1T1 CONT 312 | Viral Metagenome fragment | N/A | G | NCBI SRA |
| F1T2 CONT 1752 | Viral Metagenome fragment | N/A | G | NCBI SRA |
| F1T2 CONT 1754 | Viral Metagenome fragment | N/A | G | NCBI SRA |
| F1T2 CONT 1755 | Viral Metagenome fragment | N/A | G | NCBI SRA |
| F1T2 CONT 1759 | Viral Metagenome fragment | N/A | G | NCBI SRA |
| F1T2 CONT 1765 | Viral Metagenome fragment | N/A | G | NCBI SRA |
| F1T2 CONT 1766 | Viral Metagenome fragment | N/A | G | NCBI SRA |
| F1T2 CONT 1767 | Viral Metagenome fragment | N/A | G | NCBI SRA |
| F1T2 CONT 1768 | Viral Metagenome fragment | N/A | G | NCBI SRA |
| F1T2 CONT 1769 | Viral Metagenome fragment | N/A | G | NCBI SRA |
| F1T2 CONT 1770 | Viral Metagenome fragment | N/A | G | NCBI SRA |
| F1T2 CONT 1772 | Viral Metagenome fragment | N/A | G | NCBI SRA |
| F1T2 CONT 1773 | Viral Metagenome fragment | N/A | G | NCBI SRA |
| F1T2 CONT 1774 | Viral Metagenome fragment | N/A | G | NCBI SRA |
| F1T2 CONT 2648 | Viral Metagenome fragment | N/A | G | NCBI SRA |
| F1T2 CONT 2652 | Viral Metagenome fragment | N/A | G | NCBI SRA |
| F1T2 CONT 2653 | Viral Metagenome fragment | N/A | G | NCBI SRA |
| F1T2 CONT 2654 | Viral Metagenome fragment | N/A | G | NCBI SRA |
| F1T2 CONT 2801 | Viral Metagenome fragment | N/A | G | NCBI SRA |
| F1T2 CONT 2802 | Viral Metagenome fragment | N/A | G | NCBI SRA |
| F1T2 CONT 2803 | Viral Metagenome fragment | N/A | G | NCBI SRA |
| F1T2 CONT 2804 | Viral Metagenome fragment | N/A | G | NCBI SRA |
| F1T2 CONT 2805 | Viral Metagenome fragment | N/A | G | NCBI SRA |
| F1T2 CONT 2806 | Viral Metagenome fragment | N/A | G | NCBI SRA |
| F1T2 CONT 2807 | Viral Metagenome fragment | N/A | G | NCBI SRA |
| F1T2 CONT 2808 | Viral Metagenome fragment | N/A | G | NCBI SRA |
| F2M CONT 807 | Viral Metagenome fragment | N/A | G | NCBI SRA |
| F2M CONT 826 | Viral Metagenome fragment | N/A | G | NCBI SRA |
| F2M CONT 831 | Viral Metagenome fragment | N/A | G | NCBI SRA |
| F2M CONT 833 | Viral Metagenome fragment | N/A | G | NCBI SRA |
| F2M CONT 839 | Viral Metagenome fragment | N/A | G | NCBI SRA |
| F2M CONT 840 | Viral Metagenome fragment | N/A | G | NCBI SRA |
| F2M CONT 843 | Viral Metagenome fragment | N/A | G | NCBI SRA |
| F2M CONT 847 | Viral Metagenome fragment | N/A | G | NCBI SRA |
| F2M CONT 848 | Viral Metagenome fragment | N/A | G | NCBI SRA |
| F2M CONT 849 | Viral Metagenome fragment | N/A | G | NCBI SRA |
| F2M CONT 850 | Viral Metagenome fragment | N/A | G | NCBI SRA |
| F2M CONT 852 | Viral Metagenome fragment | N/A | G | NCBI SRA |
| F2M CONT 853 | Viral Metagenome fragment | N/A | G | NCBI SRA |
| F2M CONT 854 | Viral Metagenome fragment | N/A | G | NCBI SRA |
| F2M CONT 855 | Viral Metagenome fragment | N/A | G | NCBI SRA |
| F2M CONT 1070 | Viral Metagenome fragment | N/A | G | NCBI SRA |
| F2M CONT 1071 | Viral Metagenome fragment | N/A | G | NCBI SRA |
| F2M CONT 1072 | Viral Metagenome fragment | N/A | G | NCBI SRA |
| F2M CONT 1073 | Viral Metagenome fragment | N/A | G | NCBI SRA |
| F2M CONT 1074 | Viral Metagenome fragment | N/A | G | NCBI SRA |
| F2M CONT 1075 | Viral Metagenome fragment | N/A | G | NCBI SRA |
| F2M CONT 1322 | Viral Metagenome fragment | N/A | G | NCBI SRA |
| F2M CONT 1324 | Viral Metagenome fragment | N/A | G | NCBI SRA |
| F2M CONT 1326 | Viral Metagenome fragment | N/A | G | NCBI SRA |
| F2M CONT 1327 | Viral Metagenome fragment | N/A | G | NCBI SRA |
| F2M CONT 1328 | Viral Metagenome fragment | N/A | G | NCBI SRA |
| F2M CONT 1329 | Viral Metagenome fragment | N/A | G | NCBI SRA |
| F2M CONT 1330 | Viral Metagenome fragment | N/A | G | NCBI SRA |
| F2T1 CONT 107 | Viral Metagenome fragment | N/A | G | NCBI SRA |
| F2T1 CONT 109 | Viral Metagenome fragment | N/A | G | NCBI SRA |
| F2T1 CONT 113 | Viral Metagenome fragment | N/A | G | NCBI SRA |
| F2T1 CONT 114 | Viral Metagenome fragment | N/A | G | NCBI SRA |
| F2T1 CONT 197 | Viral Metagenome fragment | N/A | G | NCBI SRA |
| F2T1 CONT 198 | Viral Metagenome fragment | N/A | G | NCBI SRA |
| F2T1 CONT 199 | Viral Metagenome fragment | N/A | G | NCBI SRA |
| F2T1 CONT 200 | Viral Metagenome fragment | N/A | G | NCBI SRA |
| F2T1 CONT 311 | Viral Metagenome fragment | N/A | G | NCBI SRA |
| F2T1 CONT 312 | Viral Metagenome fragment | N/A | G | NCBI SRA |
| F2T2 CONT 726 | Viral Metagenome fragment | N/A | G | NCBI SRA |
| F2T2 CONT 735 | Viral Metagenome fragment | N/A | G | NCBI SRA |
| F2T2 CONT 736 | Viral Metagenome fragment | N/A | G | NCBI SRA |
| F2T2 CONT 738 | Viral Metagenome fragment | N/A | G | NCBI SRA |
| F2T2 CONT 739 | Viral Metagenome fragment | N/A | G | NCBI SRA |
| F2T2 CONT 740 | Viral Metagenome fragment | N/A | G | NCBI SRA |
| F2T2 CONT 741 | Viral Metagenome fragment | N/A | G | NCBI SRA |
| F2T2 CONT 745 | Viral Metagenome fragment | N/A | G | NCBI SRA |
| F2T2 CONT 746 | Viral Metagenome fragment | N/A | G | NCBI SRA |
| F2T2 CONT 747 | Viral Metagenome fragment | N/A | G | NCBI SRA |
| F2T2 CONT 748 | Viral Metagenome fragment | N/A | G | NCBI SRA |
| F2T2 CONT 749 | Viral Metagenome fragment | N/A | G | NCBI SRA |
| F2T2 CONT 750 | Viral Metagenome fragment | N/A | G | NCBI SRA |
| F2T2 CONT 1045 | Viral Metagenome fragment | N/A | G | NCBI SRA |
| F2T2 CONT 1051 | Viral Metagenome fragment | N/A | G | NCBI SRA |
| F2T2 CONT 1056 | Viral Metagenome fragment | N/A | G | NCBI SRA |
| F2T2 CONT 1058 | Viral Metagenome fragment | N/A | G | NCBI SRA |
| F2T2 CONT 1059 | Viral Metagenome fragment | N/A | G | NCBI SRA |
| F2T2 CONT 1060 | Viral Metagenome fragment | N/A | G | NCBI SRA |
| F3M CONT 156 | Viral Metagenome fragment | N/A | G | NCBI SRA |
| F3M CONT 157 | Viral Metagenome fragment | N/A | G | NCBI SRA |
| F3M CONT 158 | Viral Metagenome fragment | N/A | G | NCBI SRA |
| F3M CONT 159 | Viral Metagenome fragment | N/A | G | NCBI SRA |
| F3M CONT 172 | Viral Metagenome fragment | N/A | G | NCBI SRA |
| F3M CONT 173 | Viral Metagenome fragment | N/A | G | NCBI SRA |
| F3T1 CONT 54 | Viral Metagenome fragment | N/A | G | NCBI SRA |
| F3T1 CONT 55 | Viral Metagenome fragment | N/A | G | NCBI SRA |
| F3T1 CONT 79 | Viral Metagenome fragment | N/A | G | NCBI SRA |
| F3T2 CONT 326 | Viral Metagenome fragment | N/A | G | NCBI SRA |
| F3T2 CONT 333 | Viral Metagenome fragment | N/A | G | NCBI SRA |
| F3T2 CONT 335 | Viral Metagenome fragment | N/A | G | NCBI SRA |
| F3T2 CONT 343 | Viral Metagenome fragment | N/A | G | NCBI SRA |
| F3T2 CONT 344 | Viral Metagenome fragment | N/A | G | NCBI SRA |
| F3T2 CONT 348 | Viral Metagenome fragment | N/A | G | NCBI SRA |
| F3T2 CONT 349 | Viral Metagenome fragment | N/A | G | NCBI SRA |
| F3T2 CONT 676 | Viral Metagenome fragment | N/A | G | NCBI SRA |
| F3T2 CONT 683 | Viral Metagenome fragment | N/A | G | NCBI SRA |
| F3T2 CONT 685 | Viral Metagenome fragment | N/A | G | NCBI SRA |
| F3T2 CONT 693 | Viral Metagenome fragment | N/A | G | NCBI SRA |
| F3T2 CONT 694 | Viral Metagenome fragment | N/A | G | NCBI SRA |
| F3T2 CONT 698 | Viral Metagenome fragment | N/A | G | NCBI SRA |
| F3T2 CONT 699 | Viral Metagenome fragment | N/A | G | NCBI SRA |
| F4M CONT 306 | Viral Metagenome fragment | N/A | G | NCBI SRA |
| F4M CONT 309 | Viral Metagenome fragment | N/A | G | NCBI SRA |
| F4M CONT 315 | Viral Metagenome fragment | N/A | G | NCBI SRA |
| F4M CONT 316 | Viral Metagenome fragment | N/A | G | NCBI SRA |
| F4M CONT 317 | Viral Metagenome fragment | N/A | G | NCBI SRA |
| F4M CONT 318 | Viral Metagenome fragment | N/A | G | NCBI SRA |
| F4M CONT 319 | Viral Metagenome fragment | N/A | G | NCBI SRA |
| F4M CONT 418 | Viral Metagenome fragment | N/A | G | NCBI SRA |
| F4M CONT 422 | Viral Metagenome fragment | N/A | G | NCBI SRA |
| F4M CONT 423 | Viral Metagenome fragment | N/A | G | NCBI SRA |
| F4M CONT 424 | Viral Metagenome fragment | N/A | G | NCBI SRA |
| F4M CONT 425 | Viral Metagenome fragment | N/A | G | NCBI SRA |
| F4M CONT 426 | Viral Metagenome fragment | N/A | G | NCBI SRA |
| F4T1 CONT 346 | Viral Metagenome fragment | N/A | G | NCBI SRA |
| F4T1 CONT 347 | Viral Metagenome fragment | N/A | G | NCBI SRA |
| F4T1 CONT 348 | Viral Metagenome fragment | N/A | G | NCBI SRA |
| F4T1 CONT 349 | Viral Metagenome fragment | N/A | G | NCBI SRA |
| F4T1 CONT 350 | Viral Metagenome fragment | N/A | G | NCBI SRA |
| F4T1 CONT 351 | Viral Metagenome fragment | N/A | G | NCBI SRA |
| F4T1 CONT 352 | Viral Metagenome fragment | N/A | G | NCBI SRA |
| F4T1 CONT 353 | Viral Metagenome fragment | N/A | G | NCBI SRA |
| F4T1 CONT 354 | Viral Metagenome fragment | N/A | G | NCBI SRA |
| F4T1 CONT 355 | Viral Metagenome fragment | N/A | G | NCBI SRA |
| F4T1 CONT 548 | Viral Metagenome fragment | N/A | G | NCBI SRA |
| F4T1 CONT 549 | Viral Metagenome fragment | N/A | G | NCBI SRA |
| F4T1 CONT 550 | Viral Metagenome fragment | N/A | G | NCBI SRA |
| F4T1 CONT 551 | Viral Metagenome fragment | N/A | G | NCBI SRA |
| F4T1 CONT 552 | Viral Metagenome fragment | N/A | G | NCBI SRA |
| F4T1 CONT 553 | Viral Metagenome fragment | N/A | G | NCBI SRA |
| F4T1 CONT 554 | Viral Metagenome fragment | N/A | G | NCBI SRA |
| F4T1 CONT 555 | Viral Metagenome fragment | N/A | G | NCBI SRA |
| F4T1 CONT 556 | Viral Metagenome fragment | N/A | G | NCBI SRA |
| F4T1 CONT 714 | Viral Metagenome fragment | N/A | G | NCBI SRA |
| F4T1 CONT 719 | Viral Metagenome fragment | N/A | G | NCBI SRA |
| F4T1 CONT 722 | Viral Metagenome fragment | N/A | G | NCBI SRA |
| F4T1 CONT 723 | Viral Metagenome fragment | N/A | G | NCBI SRA |
| F4T1 CONT 724 | Viral Metagenome fragment | N/A | G | NCBI SRA |
| F4T1 CONT 726 | Viral Metagenome fragment | N/A | G | NCBI SRA |
| F4T1 CONT 727 | Viral Metagenome fragment | N/A | G | NCBI SRA |
| F4T2 CONT 193 | Viral Metagenome fragment | N/A | G | NCBI SRA |
| F4T2 CONT 194 | Viral Metagenome fragment | N/A | G | NCBI SRA |
| F4T2 CONT 195 | Viral Metagenome fragment | N/A | G | NCBI SRA |
| F4T2 CONT 196 | Viral Metagenome fragment | N/A | G | NCBI SRA |
| F4T2 CONT 197 | Viral Metagenome fragment | N/A | G | NCBI SRA |
| F4T2 CONT 198 | Viral Metagenome fragment | N/A | G | NCBI SRA |
| F4T2 CONT 199 | Viral Metagenome fragment | N/A | G | NCBI SRA |
| F4T2 CONT 200 | Viral Metagenome fragment | N/A | G | NCBI SRA |
| F4T2 CONT 201 | Viral Metagenome fragment | N/A | G | NCBI SRA |
| F4T2 CONT 388 | Viral Metagenome fragment | N/A | G | NCBI SRA |
| F4T2 CONT 389 | Viral Metagenome fragment | N/A | G | NCBI SRA |
| F4T2 CONT 390 | Viral Metagenome fragment | N/A | G | NCBI SRA |
| Bacteroides vulgatus ATCC 8482 chromosome complete genome | Bacterial Chromosome | complete | - | NCBI |
| Bacteroides fragilis YCH46 chromosome complete genome | Bacterial Chromosome | complete | - | NCBI |
| Bacteroides fragilis NCTC 9343 chromosome complete genome | Bacterial Chromosome | complete | - | NCBI |
| Bacteroides thetaiotaomicron VPI-5482 | Bacterial Chromosome | complete | - | NCBI |
| Bacteroides helcogenes P 36-108 chromosome complete genome | Bacterial Chromosome | complete | - | NCBI |
| Bacteroides salanitronis DSM 18170 chromosome complete genome | Bacterial Chromosome | complete | - | NCBI |
| Bacteroides sp 3_2_5_1 | Bacterial Chromosome | Draft | - | Broad Inst |
| Bacteroides fragilis 3_1_12_1 | Bacterial Chromosome | Draft | - | Broad Inst |
| Bacteroides sp. 2_1_16_1 | Bacterial Chromosome | Draft | - | Broad Inst |
| Bacteroides finegoldii 1_0_1 | Bacterial Chromosome | Draft | - | Broad Inst |
| Bacteroides sp. 1_1_6_1 | Bacterial Chromosome | Draft | - | Broad Inst |
| Bacteroides thetaiotaomicron 3731-4 0 | Bacterial Chromosome | Draft | - | WUGI |
| Bacteroides thetaiotaomicron 3330-6 0 | Bacterial Chromosome | Draft | - | WUGI |
| Bacteroides sp. 1_1_14_1 | Bacterial Chromosome | Draft | - | Broad Inst |
| Bacteroides sp. 2_2_4_1 | Bacterial Chromosome | Draft | - | Broad Inst |
| Bacteroides sp. d22_1 | Bacterial Chromosome | Draft | - | Broad Inst |
| Bacteroides sp. 3_1_23_1 | Bacterial Chromosome | Draft | - | Broad Inst |
| Bacteroides sp. d1_1 | Bacterial Chromosome | Draft | - | Broad Inst |
| Bacteriodes cellulolyticus 1_0 | Bacterial Chromosome | Draft | - | WUGI |
| Bacteroides sp. 2_1_22_1 | Bacterial Chromosome | Draft | - | Broad Inst |
| Bacteroides ovatus | Bacterial Chromosome | Draft | - | WUGI |
| Bacteroides novel WH2 6_0 | Bacterial Chromosome | Draft | - | WUGI |
| Bacteroides sp. d2_1 | Bacterial Chromosome | Draft | - | Broad Inst |
| Bacteroides intestinalis 2_0 | Bacterial Chromosome | Draft | - | WUGI |
| Bacteroides sp. 20_3_1 | Bacterial Chromosome | Draft | - | Broad Inst |
| Bacteriodes caccae 4_0_1 | Bacterial Chromosome | Draft | - | WUGI |
| Bacteroides sp. 2_1_7_1 | Bacterial Chromosome | Draft | - | Broad Inst |
| Bacteroides sp 4_3_47faa | Bacterial Chromosome | Draft | - | Broad Inst |
| Bacteroides sp. 3_1_19_1 | Bacterial Chromosome | Draft | - | Broad Inst |
| Bacteroides sp. 2_1_33b 1 | Bacterial Chromosome | Draft | - | Broad Inst |
| Bacteroides sp. 3_1_40a 1 | Bacterial Chromosome | Draft | - | Broad Inst |
| Bacteroides eggerthii 1 _2_48faa | Bacterial Chromosome | Draft | - | Broad Inst |
| Bacteroides sp. d4_1 | Bacterial Chromosome | Draft | - | WUGI |
| Bacteriodes eggerthii 1_0 | Bacterial Chromosome | Draft | - | WUGI |
| Bacteroides vulgatus 1_0 | Bacterial Chromosome | Draft | - | WUGI |
| Bacteriodes dorei 1_0 | Bacterial Chromosome | Draft | - | WUGI |
| Bacteroides sp. 3_1_23_1 | Bacterial Chromosome | Draft | - | Broad Inst |
| Bacteroides sp. 9_1_42faa | Bacterial Chromosome | Draft | - | Broad Inst |
| Bacteriodes distasonis 1_0 | Bacterial Chromosome | Draft | - | WUGI |
| Bacteroides stercoris 2_0_1 | Bacterial Chromosome | Draft | - | WUGI |
| Bacteriodes coprocola 2_0_1 | Bacterial Chromosome | Draft | - | WUGI |
| Bacteroides sp. 4_1_36_1 | Bacterial Chromosome | Draft | - | Broad Inst |
| Bacteroides uniformis 3_0_1 | Bacterial Chromosome | Draft | - | WUGI |
| Bacteriodes coprophilus 1_0 | Bacterial Chromosome | Draft | - | WUGI |
| Bacteroides plebius 2_0_1 | Bacterial Chromosome | Draft | - | WUGI |
| Bacteroidetes oral taxon 274 str. f0058_1 | Bacterial Chromosome | Draft | - | Broad Inst |
| Bacteroides pectinophilus 1_0_1 | Bacterial Chromosome | Draft | - | WUGI |
| Bacteriodes capillosarus 2_0_1 | Bacterial Chromosome | Draft | - | WUGI |
